# Multiomics Analysis of the PHLDA Gene Family in Different Cancers and Their Clinical Prognostic Value

**DOI:** 10.3390/cimb46060328

**Published:** 2024-05-30

**Authors:** Safia Iqbal, Md. Rezaul Karim, Shahnawaz Mohammad, Ramya Mathiyalagan, Md. Niaj Morshed, Deok-Chun Yang, Hyocheol Bae, Esrat Jahan Rupa, Dong Uk Yang

**Affiliations:** 1 Department of Biopharmaceutical Biotechnology, College of Life Science, Kyung Hee University, Yongin-si 17104, Gyeonggi-do, Republic of Korea; safiadorin@khu.ac.kr (S.I.); rezaulshimul@khu.ac.kr (M.R.K.); niajmorshed96@khu.ac.kr (M.N.M.); dcyang@khu.ac.kr (D.-C.Y.); 2 Graduate School of Biotechnology, College of Life Science, Kyung Hee University, Yongin-si 17104, Gyeonggi-do, Republic of Korea; shnwzmohd@yahoo.com (S.M.); ramyabinfo@gmail.com (R.M.); 3 Department of Oriental Medicinal Biotechnology, College of Life Sciences, Kyung Hee University, Yongin-si 17104, Gyeonggi-do, Republic of Korea; bhc7@khu.ac.kr; 4 College of Korean Medicine, Woosuk University, Wanju-gun 55338, Jeollabuk-do, Republic of Korea

**Keywords:** multiomics, PHLDA (pleckstrin homology-like domain family) gene, cancer biomarker, gene ontology (GO), gene pathway

## Abstract

The PHLDA (pleckstrin homology-like domain family) gene family is popularly known as a potential biomarker for cancer identification, and members of the PHLDA family have become considered potentially viable targets for cancer treatments. The PHLDA gene family consists of PHLDA1, PHLDA2, and PHLDA3. The predictive significance of PHLDA genes in cancer remains unclear. To determine the role of pleckstrin as a prognostic biomarker in human cancers, we conducted a systematic multiomics investigation. Through various survival analyses, pleckstrin expression was evaluated, and their predictive significance in human tumors was discovered using a variety of online platforms. By analyzing the protein–protein interactions, we also chose a collection of well-known functional protein partners for pleckstrin. Investigations were also carried out on the relationship between pleckstrins and other cancers regarding mutations and copy number alterations. The cumulative impact of pleckstrin and their associated genes on various cancers, Gene Ontology (GO), and pathway analyses were used for their evaluation. Thus, the expression profiles of PHLDA family members and their prognosis in various cancers may be revealed by this study. During this multiomics analysis, we found that among the PHLDA family, PHLDA1 may be a therapeutic target for several cancers, including kidney, colon, and brain cancer, while PHLDA2 can be a therapeutic target for cancers of the colon, esophagus, and pancreas. Additionally, PHLDA3 may be a useful therapeutic target for ovarian, renal, and gastric cancer.

## 1. Introduction

One of the main causes of death in the world is cancer. The incidence of cancer and associated survival rates vary significantly depending on the patient’s nationality, ethnicity, and socioeconomic factors [1]. Consequently, a highly desired goal of cancer research worldwide is the creation of more efficient biomarkers for prognosis. Cancer has a variety of impacts on intracellular metabolism that allow cancer cells to grow uncontrollably, adapt to the tumor microenvironment, and alter the metabolism of normal tissue [2]. Cancer metabolism refers to all of these changes in cellular metabolic pathways that help cancer cells develop and maintain their malignant characteristics [3]. Pleckstrin homology domain family A (PHLDA) genes are a family of tumor oncogenes that play crucial roles in the cellular processes of cancer [4,5]. The pleckstrin homology-like domain (PHLD) protein is a convenient protein that contains the P^H^ domain, and in the phosphorylated head group of phosphatidylinositol 4,5-bisphosphate (PtdI(4,5)P2) lipids, it binds with significant in vitro specificity [6]. There are three members of the PHLDA gene family, including PHLDA1, PHLDA2, and PHLDA3. All three genes code proteins with P^H^ domains. Due to their substantial similarity within their P^H^ domains, these proteins can be detected via immunohistochemical screening with different sensitivities and specificities [7]. One coding exon and one noncoding exon are separated by a brief intron in the genes belonging to the PHLDA family [8]. Several studies have established the involvement of the PHLDA family of tumor oncogenes in critical elements of cancer cell processes, such as competition with Akt signaling [9], the repression of growth factor signaling [10], and the activation of apoptosis [8]. The PHLDA family can activate the EMT (epithelial–mesenchymal transition) signaling pathway and promote the occurrence and development of cancer [11]. There is a tiny fraction of tumor cells that are incredibly resistant to conventional therapies; this fraction is typically less than 1%. These cells are known as cancer-initiating cells or cancer stem cells. Cancer stem cells have a strong proliferative capacity, self-renewal potential, and multipotency, which contribute to tumor invasion, metastasis, and recurrence after therapy. Breast, colon, lung, prostate, liver, melanoma, leukemia, head and neck, ovarian, pancreatic, and brain tumors are a few of the cancers in which the presence of CSCs has been documented [12]. Two members from the pleckstrin family, PHLDA1 [13] and PHLDA3 [14], are considered valuable markers of hair follicle (HF) stem cells. These HF cells exhibit (EMT) cells [14] and should be linked to the stemness of tumor-initiating CSCs (cancer stem cells) and intrinsic resistance to standard therapies [15]. It is important to find appropriate markers for detecting CSCs to create targeted therapeutics. PHLDA1 was also discovered to be expressed in relation to a variety of cancers, including lung, bladder, liver, and brain cancers [8]. PHLDA1 is a potential epithelial stem cell marker that promotes colon cancer cell motility and proliferation in the small and large intestines of humans [16]. The PHLDA1 gene, which was first identified in T-cell hybridomas, encodes a protein with a high proline and histidine content and is evolutionarily conserved [17]. PHLDA1 may have a role in a variety of biological processes, including cell differentiation, cell proliferation, cell death, cancer metastasis, and epithelial–mesenchymal transition; it also has tumor stem cell properties [18]. It was revealed that PHLDA1 mRNA expression was higher, which leads to a higher risk of distant metastasis in estrogen receptor (ER)-positive breast cancers, but PHLDA1 exerts the opposite effects on breast cancers that are ER-negative [19]. Reduced PHLDA1 expression has been observed in gastric adenocarcinoma, breast carcinoma, oral carcinoma [8], and melanoma [20]. Additionally, in mice xenograft models, lower PHLDA1 expression slows the progression of overall survival metastasis, while high PHLDA1 expression is linked to high tumor progression in osteosarcoma cells [21]. Although there has been great advancement over the past decade in the detection and treatment of cancer development and prognosis, there is not enough research on the relationship between PHLDA1 expression and prognosis in some tumors.

PHLDA2 is one of the first genes associated with apoptosis that is controlled by genomic imprinting [22] and has been linked to early growth and development. PHLDA2 shares a pleckstrin homology domain with PHLDA1 and PHLDA3 (PH domain). The expression of PHLDA2 was found to be elevated in colorectal cancer [23]. The migration and proliferation of breast cancer cells were significantly reduced when the PHLDA2 gene was silenced [23]. PHLDA2 enhances pancreatic ductal adenocarcinoma tumor growth [24], and its downregulation is seen in some human cancers, including osteosarcoma; hence, it has tumor-suppressing properties [25]. It acts as an oncogene in pancreatic ductal carcinoma and breast cancer, which may be connected to the origin of tumor tissue [26]. Despite all of these approaches, there is still little research on PHLDA2 expression in cancer and its relevance to clinical outcomes. Moreover, PHLDA2 may be used as a possible biomarker of AKT pathway activity. Despite the fact that PHLDA2 and PHLDA3 share a lot of similarities, PHLDA3 functions as a protein that solely has P^H^ domains and interacts with another protein that also contains P^H^ domains. PHLDA3 localizes on the cell membrane and binds to a variety of phosphatidylinositol phosphates (PIPs) via this domain [27]. The function of PHLDA3 might be a valuable prognostic indicator. Further, PHLDA3 has been linked to a variety of cancers, including lung endocrine cancers, pancreatic neuroendocrine cancers, primary breast cancers, and prostate carcinomas, based on the traits of its tumorigenesis-related cell activities. A study discovered that the largest increase in PHLDA3 was seen in injured tubules as kidney injury worsened [28]. PHLDA3 is connected to the control of acute liver damage and acute kidney injury, according to several pieces of research [28]. The prognosis of cardiovascular diseases can be improved in cases of PHLDA3 deficiency, suggesting that it may play a role in heart failure and pathological cardiac hypertrophy [29]. However, it is still unknown how PHLDA3 affects heart failure and pathological cardiac hypertrophy. PHLDA3 may have a function in tumor suppression as the loss of the PHLDA3 chromosomal locus is frequently seen in primary lung tumors [30]. Additionally, to the best of our knowledge, data mining techniques have never been used to comprehensively explore the pleckstrin genes in various malignancies. However, related research discovered that the P^H^ domain can promote the migration and proliferation of tumor cells [31]. The integration of multiomics data offers a way of studying the biology underlying complicated diseases like cancer. Therefore, we evaluated the significance of pleckstrin expression in various human malignancies using a range of online bioinformatics platforms and tools in this context. We also investigated the clinical significance of the PHLDA1, PHLDA2, and PHLDA3 genes, as well as their patterns of expression, mutation, and copy number variation in human malignancies. In the current study, we assessed the expression of the PHLDA gene family in cancers to support the clinical relevance of PHLDA genes as prospective agnostic biomarkers. Further, we focused on the interaction partners and genes that were co-expressed with PHLDA1, PHLDA2, and PHLDA3 in diverse malignancies to assess the likely underlying signaling pathways involved. According to the data, various tumor tissues and cell types may respond differently to members of the PHLDA family, and their particular outcomes are dependent on the cancer cells [32]. This finding provides valuable knowledge that will contribute to establishing anti-cancer therapies and identifying new targetable treatment pathways for patients across various cancers that target cancer stem cells.

## 2. Materials and Methods

### 2.1. Oncomine Analysis

The mRNA expression levels of PHLDA1, PHLDA2, and PHLDA3 relating to various cancer types were retrieved through the online Oncomine database (https://www.oncomine.org/resource/login.html) (accessed on 3 April 2023) [33]. The Oncomine platform is an online cancer microarray database that allows genome-wide expression analyses. The threshold conditions were as follows: fold-change > 2, significant *p*-value 0.01, and gene ranking in the top 10%. Supplementary Tables S1–S3 summarize the details of the analyses.

### 2.2. Analysis of the Expression of Gene and Mutation by Using cBioPortal

Interactive visualization and analysis of multidimensional cancer genomics datasets were performed using the cBioPortal (v. 6.0.5) [34]. Genetic changes within specific genes across accessible samples are possible to obtain via the query interface and individualized data storage. cBioPortal was used to examine expression pattern, missense mutation, deep deletion, copy number alteration (CNA), and RNA sequencing data in this work. Using the default settings, the main search criteria comprised RNA sequencing data, copy number alterations (CNAs) from GISTIC, and changes (amplifications, deep deletions, and missense mutations). We employed RNA sequencing data as the primary focus of our secondary search.

### 2.3. Prognosis Analysis Using PrognoScan

The meta-analysis to determine the prognostic value of several genes was carried out using PrognoScan (http://dna00.bio.kyutech.ac.jp/PrognoScan/) (accessed on 12 April 2023) [35]. In many patient datasets from cancer microarrays, this web-based tool helps with the examination of the relationship between gene expression and patient prognosis. Several studies have looked for the correlation between PHLDA expression and survival in several cancer types using this tool. The significance level was set at adjusted to a Cox *p*-value of less than 0.05. These findings are shown in Supplementary Tables S3–S5.

### 2.4. mRNA Expression Profiling with GEPIA

One of the developed interactive online tools for evaluating RNA sequencing data is Gene Expression Profiling Interactive Analysis (GEPIA; available at http://gepia.cancer-pku.cn/) (accessed on 18 April 2023) [36]. The Genotype-Tissue Expression (GTEx) project and the Cancer Genome Atlas (TCGA) are the foundations for the data analysis carried out using GEPIA. It offers access to a vast array of information from the Genotype-Tissue Expression (GTEx) project and The Cancer Genome Atlas (TCGA), including 9736 tumor samples and 8587 normal samples. The box plots tool available in GEPIA was used to conduct tumor/normal differential expression analysis for PHLDA1, PHLDA2, and PHLDA3 in various malignancies. In addition, GEPIA provides additional customizable features, including correlation and patient survival analysis.

### 2.5. Survival Data Analysis Applying Kaplan–Meier Plotter

A web-based program called the Kaplan–Meier Plotter (http://kmplot.com/analysis/) (accessed on 5 May 2023) [37] was used to examine whether different genes affect cancer patients’ survival rates. Data from 30k+ samples of 21 tumor types, including breast, ovarian, lung, and gastric cancer, were used to analyze the effect of 70,632 genes on survival using the Kaplan–Meier Plotter. Meta-analysis-based biomarker assessment is the major goal of this tool. This tool separates patient samples into pairs of groups based on various quantiles of biomarker expression to assess the prognostic significance of a particular gene. The Kaplan-Meier Plotter was used in this study to assess the correlations between the expression of pleckstrin and the survival of patients with cancers of interest. The program divides patient samples into groups based on different quantiles of biomarker expression in order to assess the prognostic significance of a given gene. To compare the two patient groups and determine the log-rank *p*-value and the hazard ratio, along with 95% confidence intervals, Kaplan-Meier survival plots were created.

### 2.6. R2 Platform for Correlation and Survival Analysis

R2 (http://r2platform.com) (accessed on 14 May 2023) is a platform for genomics analysis and visualization that includes a database and a web interface with a set of analysis tools [38]. The best cut-off values for PHLDA1, PHLDA2, and PHLDA3 were determined using the Kaplan Scan feature and the *p*-value from a log-rank test of the relevant cancers. A list of genes correlated with PHLDA1, PHLDA2, and PHLDA3 was also found and imported using the R2 program. The binary heat map was utilized to display clustering based on “good” vs. “bad” prognoses in addition to the Kaplan Scan tool. We also examined the publicly accessible TCGA information on the R2 website to find genes linked to PHLDA1, PHLDA2 and PHLDA3 genes. Using the functional enrichment analysis program Venny 2.1 (https://bioinfogp.cnb.csic.es/tools/venny/) (accessed on 27 May 2023), the imported data were then used to identify the pleckstrin-correlated genes common to all relevant tumors. Using the Protein Analysis Through Evolutionary Relationships (PANTHER) tool (http://pantherdb.org/) (accessed on 29 May 2023), gene ontology and pathway analyses were carried out to identify how these commonly related genes together influence signaling pathways [39]. This is an online system that uses several ontologies, such as molecular function, biological process, cellular components, and route, to categorize proteins (and their genes).

### 2.7. Protein–Protein Interaction (PPI) Analysis Using GeneMANIA

GeneMANIA (https://genemania.org) (accessed on 8 June 2023) is an online tool that evaluates gene lists and genes based on functional assessments and gives information about hypothesized gene functions [40]. It includes a significant collection of functional association data, including information on pathways, co-expression, and the interactions between proteins and genes. The prediction output takes the form of a network, where nodes stand in for genes and links for networks, which illustrates the associations between the genes in the list. As queries, PHLDA1, PHLDA2, and PHLDA3 were used to predict PPIs using the GeneMANIA analytic program.

### 2.8. Statistical Analysis

Expression data were retrieved from the Oncomine, cBioPortal, and GEPIA databases. Two groups (normal vs. cancer) were analyzed using an unpaired *t*-test, where a significance level of *p* < 0.05 was used. The R2: Kaplan–Meier Scanner and Kaplan–Meier Plotter databases were used to derive survival curves. *p*-values from a log-rank test are displayed with all results for every test. Additionally, data on clinical outcomes were obtained from the Kaplan–Meier Plotter database.

## 3. Results

**Figure 1:**
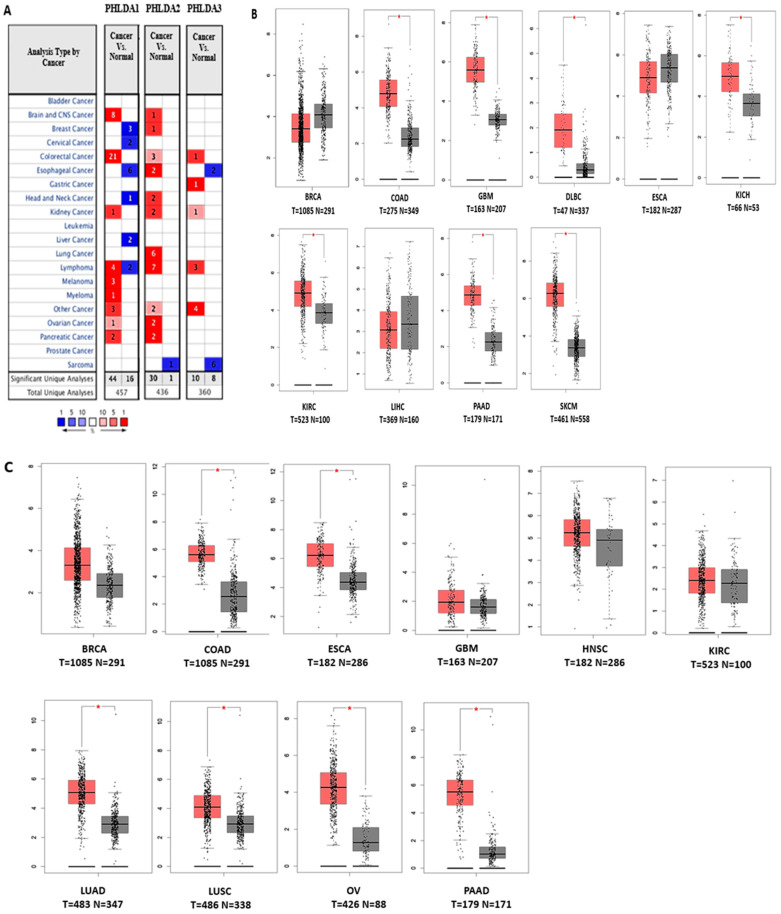

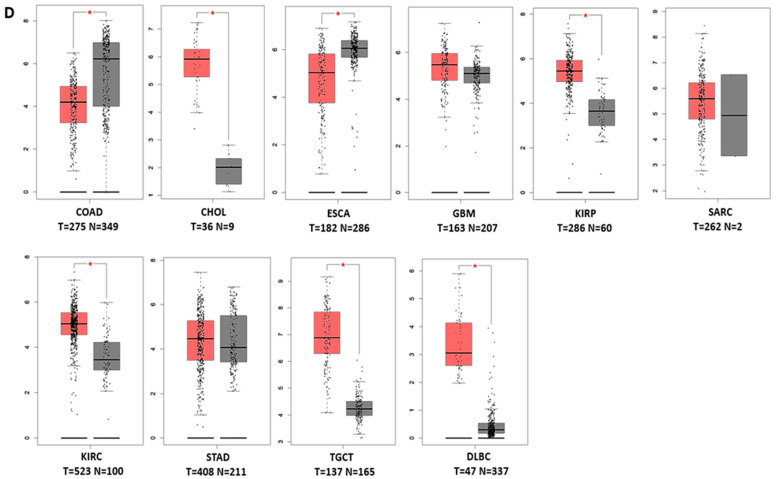
PHLDA1, PHLDA2, and PHLDA3 transcription levels in various types of cancer (Oncomine and TCGA databases). (**A**). The number of datasets where the overexpression (red) or underexpression (blue) of PHLDA1, PHLDA2, and PHLDA3 mRNA (cancer vs. corresponding normal tissue) is statistically significant (*p* ≤ 0.01) is shown in this graphic, which was created using Oncomine (available at https://www.oncomine.org/resource/login.html) (accessed on 3 April 2023). The threshold was established with the following parameters: 10% gene ranking, a fold change of 2, and a *p*-value of 1 × 10^−5^. The numbers in the boxes show the number of analyses that met these requirements (**B**–**D**). (**B**) PHLDA1 expression in The Cancer Genome Atlas (TCGA) database. Using TCGA data from GEPIA, box plots illustrating PHLDA1 mRNA expression in various tumor (T) and normal (N) tissues are shown. The following parameters were used when designing the threshold: *p*-value is 0.01, and the fold change is 2. (Abbreviations: COAD, colon adenocarcinoma; GBM, brain glioblastoma; LUAD, lung adenocarcinoma; DLBCL, diffuse large B cell lymphoma; KICH, kidney chromophobe; KIRC, kidney renal clear cell carcinoma; SKCM, skin cutaneous melanoma; PAAD, pancreatic adenocarcinoma; LIHC, liver hepatocellular carcinoma; BRCA, invasive breast carcinoma; ESCA, esophageal carcinoma) (**C**). PHLDA2 gene expression in The Cancer Genome Atlas (TCGA) database. Box plots using data from the TCGA database via the GEPIA website demonstrating the expression of PHLDA2 mRNA in various tumor (T) and normal (N) tissues. The following parameters were used to design the threshold: *p*-value = 0.01, fold change = 2. (Abbreviations: BRCA, invasive breast carcinoma; COAD, colon adenocarcinoma; ESCA, esophageal carcinoma; GBM, glioblastoma multiforme; HNSC, head and neck squamous cell carcinoma; KIRC, kidney renal clear cell carcinoma; LUAD, lung adenocarcinoma; LUSC, lung squamous cell carcinoma; OV, ovarian cancer; PAAD, pancreatic adenocarcinoma) (**D**). Expression of the PHLDA3 gene in The Cancer Genome Atlas (TCGA) database. Box plots displaying the expression of PHLDA3 mRNA in various tumor (T) and normal (N) tissues were created using information from the TCGA database via the GEPIA website. The threshold was created using the parameters *p*-value = 0.01 and fold change = 2. (Abbreviations: CHOL, cholangiocarcinoma; GBM, glioblastoma multiforme; KIRP, kidney renal papillary cell carcinoma; KIRC, kidney renal clear cell carcinoma; TGCT, Tenosynovial giant cell tumour; COAD, colon adenocarcinoma; DLBCL, diffuse large B cell lymphoma; ESCA, esophageal carcinoma; SARC, sarcoma; STAD, stomach adenocarcinoma).

We examined the contribution of the pleckstrin homology-like domain family to the growth of human malignancies using the Oncomine database. Via the visualization capabilities supplied by the Oncomine database, the transcription levels in cancers were compared with those seen in normal tissues. Without adjusting any filter settings, the numerous underlying threshold values were as follows: *p*-value, 0.01; fold change, 2; and gene ranking, 10%. In relation to these parameters, we discovered that, in comparison to the expression levels in normal tissues, pleckstrin family member A was overexpressed in some cancer tissues and downregulated in others. These results suggest that pleckstrin may have either an oncogenic or an anti-oncogenic function depending on the type of cancer (Figure 1A). The TCGA database, a platform containing a substantial collection of RNA sequencing studies, helps in deriving the molecular causes of cancer. We also conducted additional research on the mRNA levels of pleckstrin in various cancer types using TCGA data accessed through cBioPortal (Figure 1B). Further analyses used in pleckstrin analyses are provided below.

### 3.1. Transcript Expression Analysis of PHLDA in Various Cancers

**Figure 2:**
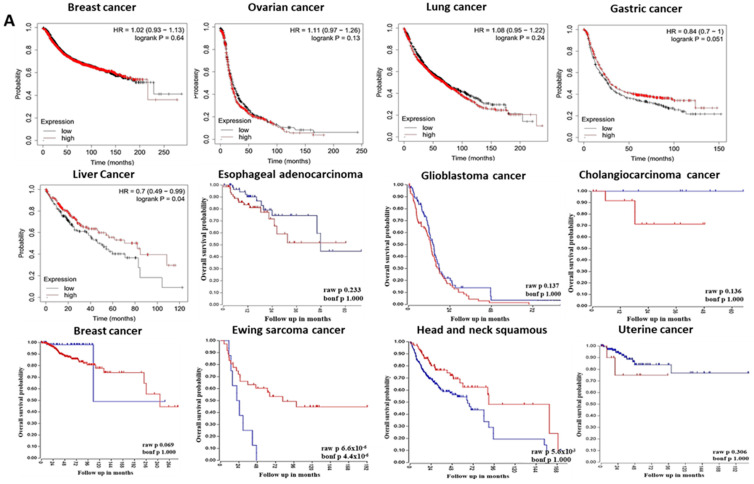

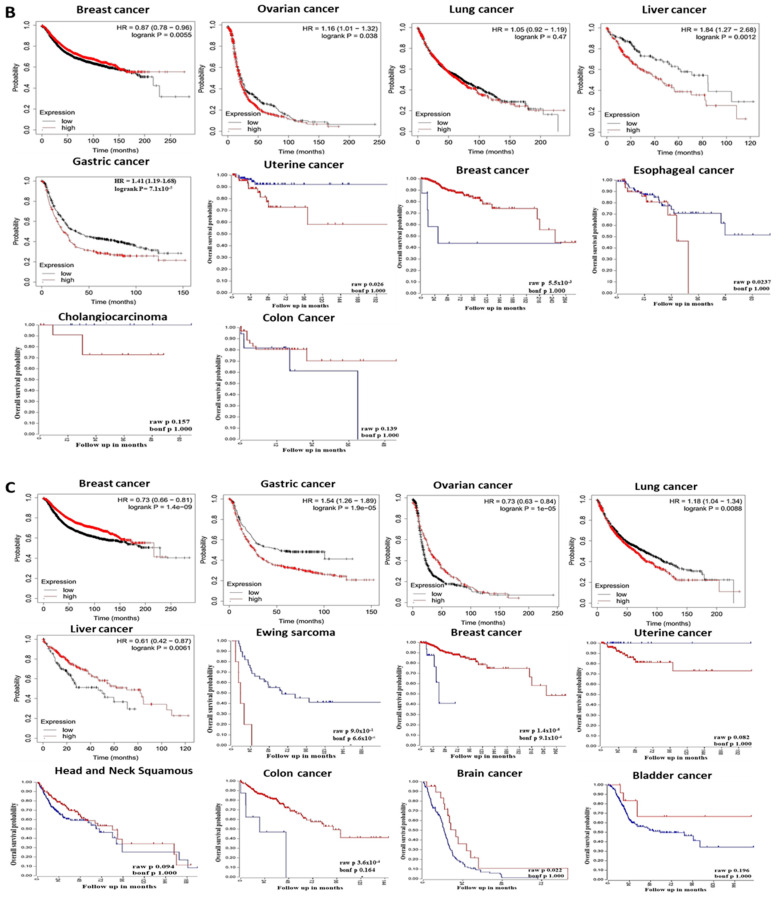
Correlation of PHLDA1, PHLDA2, and PHLDA3 expression with the prognosis of various cancers. (**A**). Correlation of PHLDA1 expression with the prognosis of various cancers (R2: Kaplan-Meier Scanner; Kaplan-Meier Plotter). Patients with high (red) and low (blue) PHLDA1 expression were compared on survival curves using breast, ovarian, lung, gastric, and liver data from Kaplan-Meier Plotter; esophageal cancer, brain, cholangiocarcinoma, breast, Ewing sarcoma, head, and neck squamous, uterine data from R2: Kaplan-Meier Scanner. Cox *p*-value threshold < 0.05. For PHLDA1, gene expression was not statistically significant for breast, ovarian, lung, esophageal, glioblastoma, cholangiocarcinoma, head and neck squamous, and uterine cancer (**B**). Correlation of PHLDA2 expression with the prognosis of various cancers (R2: Kaplan–Meier Scanner; Kaplan-Meier Plotter). Survival curves comparing patients with high (red) and low (blue) PHLDA2 expression were plotted using breast, ovarian, lung, liver, and gastric data from Kaplan-Meier Plotter; uterine cancer, breast, esophageal, cholangiocarcinoma, and colon cancer data from R2: Kaplan–Meier Scanner. Cox *p*-value threshold < 0.05. For PHLDA2, gene expression was not statistically significant for cholangiocarcinoma and colon cancer (**C**). Correlation of PHLDA3 expression with the prognosis of various cancers (R2: Kaplan-Meier Scanner, Kaplan-Meier Plotter). Survival curves comparing patients with high (red) and low (blue) PHLDA3 expression were plotted using breast, gastric, ovarian, lung, and liver data from Kaplan-Meier Plotter; Ewing sarcoma cancer, breast, uterine, head, and neck squamous, colon, brain cancer, and bladder data from R2: Kaplan-Meier Scanner. *p* < 0.05 represents statistical significance. For PHLDA3, gene expression was not statistically significant for lung, liver, and head and neck squamous cancer.

We applied online analytical tools and databases to assess pleckstrin mRNA expression levels in normal and cancerous tissue types. Using the web tool GEPIA, we conducted PHLDA1 single-gene analysis. These results are shown in Figure 2A (patients with high (red) and low (blue) PHLDA1 overexpression in the colon, brain, lymphoma, kidney, pancreatic, and skin cancers and PHLDA1 downregulation in the breast, esophageal, and liver (Table S1). When compared to the pattern of PHLDA1 expression, the pattern of PHLDA2 expression in various tumors was noticeably different. In contrast to normal tissues, we found that PHLDA2 is significantly overexpressed in the colon, breast, esophageal, brain, kidney, lung, ovaries, and pancreas (Figure 2B; Table S2). The pattern of PHLDA3 is overexpressed in cholangiocarcinoma, brain cancers, kidney cancers, testicular cancer, and lymphoma; however, it has lower expression in colon, esophageal, sarcoma, and stomach cancers (Figure 2C). These findings can be considered relevant due to the fact that the pleckstrin expression status was verified across several databases. The systematic study performed here evaluated pleckstrin family member A and mRNA expression status across a variety of cancer types. Furthermore, other research has provided support for these findings regarding PHLDA1, PHLDA2, and PHLDA3 expression. For example, PHLDA1 overexpression has been found in the colon [41], skin [20], breast [42], lung, and stomach [8]. In contrast, our analysis showed that PHLDA2 is overexpressed in lung and colorectal cancers, and it may also be downregulated in pancreatic carcinoma. On the other hand, PHLDA1 expression is also a significant indicator of poor prognoses in breast cancer patients [8]. For PHLDA3, from a research article, it is clear that this gene is upregulated in kidney cancer [28] and that this gene is mutated in lung cancer [43]. PHLDA3 indicates poor prognosis in patients with esophageal squamous cell carcinoma. We subsequently investigated the extent to which pleckstrin family member A expression is connected to prognosis.

### 3.2. The Prognostic Value of Pleckstrin Family Member A

PrognoScan, R2, and the Kaplan–Meier Plotter were a few of the web tools used to assess the relationship between pleckstrin gene expression and clinical prognosis in this investigation. Moreover, a correlation was shown between PHLDA1 overexpression and a negative prognosis in breast, ovary, lung, brain, and cholangiocarcinoma cancers 3) (Figure 2), as analyzed using PrognoScan and the Kaplan–Meier Plotter (Table S4) databases. Furthermore, gastric, liver, and sarcoma tumors were associated with reduced PHLDA1 expression (Figure 2A). Surprisingly, based on this study, the website tool GEPIA also revealed the low expression of PHLDA1 in liver cancer (Figure 2A [LIHC]). These findings suggested that PHLDA1 is a tumor-suppressing gene for gastric and liver cancers but an oncogene for breast, ovarian, and lung cancers. However, it was unclear how PHLDA1 expression correlated with breast cancer survival (Figure 2A). For example, an analysis of the GEO dataset using the prognostic database (Table S4) showed the overall survival of a patient with breast cancer and lung cancer in relation to PHLDA1 gene expression. Similarly, data derived from the R2 platform showed that high levels of PHLDA1 expression were linked to low overall survival (OS) in patients with esophageal and cholangiocarcinoma cancer (Figure 2A). The other study claims that the relationship between PHLDA2 expression and breast cancer patient survival is ambiguous and inconsistent (Figure 2B). The disparity in survival statistics between the R2: Kaplan–Meier Scanner and the Kaplan–Meier Plotter could be caused by an insufficient number of studies and reports.

The analysis of the PrognoScan database showed PHLDA2 expression in patients with several cancers (Table S5). We observed that high levels of PHLDA2 expression were associated with poor survival in ovarian, liver, gastric, uterine, esophageal, and cholangiocarcinoma cancer patients (Figure 2B) when using the R2 platform. In contrast, low PHLDA2 expression was associated with poor clinical outcomes in patients with breast and colon cancer (Figure 2B).

In gastric, ovarian, sarcoma, and uterine cancer, we found a direct link between PHLDA3 overexpression and poor patient survival (Figure 2C). Surprisingly, the Oncomine database revealed elevated PHLDA3 levels in gastric cancer depending on the samples and relevant studies analyzed (Figure 1A). However, there was a strong positive correlation between the low level of PHLDA2 expression and high survival in these forms of cancer. Moreover, liver and colon cancer survival rates were positively linked with upregulated PHLDA3 levels (Figure 2C). In these cancers, a decline in the expression levels of pleckstrin family members was positively correlated with poor survival. Moreover, in general, any deviation in PHLDA3 expression, when compared to the expression mark observed in normal tissues, was associated with poor prognosis in breast and brain cancer (Figure 2C).

Based on information from the PrognoScan database, the predictive significance of PHLDA1, PHLDA2, and PHLDA3 expression levels for various cancer patients was also evaluated (Tables S4–S6). In summary, a comprehensive analysis of survival data from a range of online resources highlighted the oncogenic role of PHLDA1 in brain cancer (Figure 1A,B) and pancreatic cancer (Figure 1A and Figure 2A). In contrast, unlike the case of PHLDA1, the oncogenic role of PHLDA2 was evident in lung, ovarian, and pancreatic malignancies (Figure 1A,C and Figure 2B). The oncogenic role of PHLDA3 was upregulated in gastric cancer (Figure 1A and Figure 2C). However, the role of PHLDA3 in colon cancer was not clear. According to these findings, PHLDA1, PHLDA2, and PHLDA3 may partially co-express to control the prognosis of cancer.

Overall, our assessments of patient survival performed on a variety of platforms, such as R2, Oncomine, and PrognoScan, highlighted the oncogenic significance of PHLDA1 in brain and pancreatic cancer. The oncogenic significance of PHLDA2 in ovarian and pancreatic cancer, however, revealed that other factors should be considered when determining the prognosis of cancer. Nevertheless, PHLDA3 could play an oncogenic role in gastric cancer. Our study of the underlying mechanism of cancer prognosis concerning pleckstrin expression may be aided by the finding that the patterns of PHLDA1, PHLDA2, and PHLDA3 co-expression regulated the clinical outcomes of individuals with certain types of cancer. Furthermore, the interaction between these three pleckstrin family member A may be associated with the progression of various types of cancers.

### 3.3. Predicting Protein–Protein Interaction of Pleckstrin Family Member A

Using the cBioPortal, we independently analyzed the genetic changes in PHLDA1, PHLDA2, and PHLDA3 in distinct cancer types and then provided a comparative report regarding the functional protein partners of glutaminases obtained using PPI network analysis.

**Figure 3:**
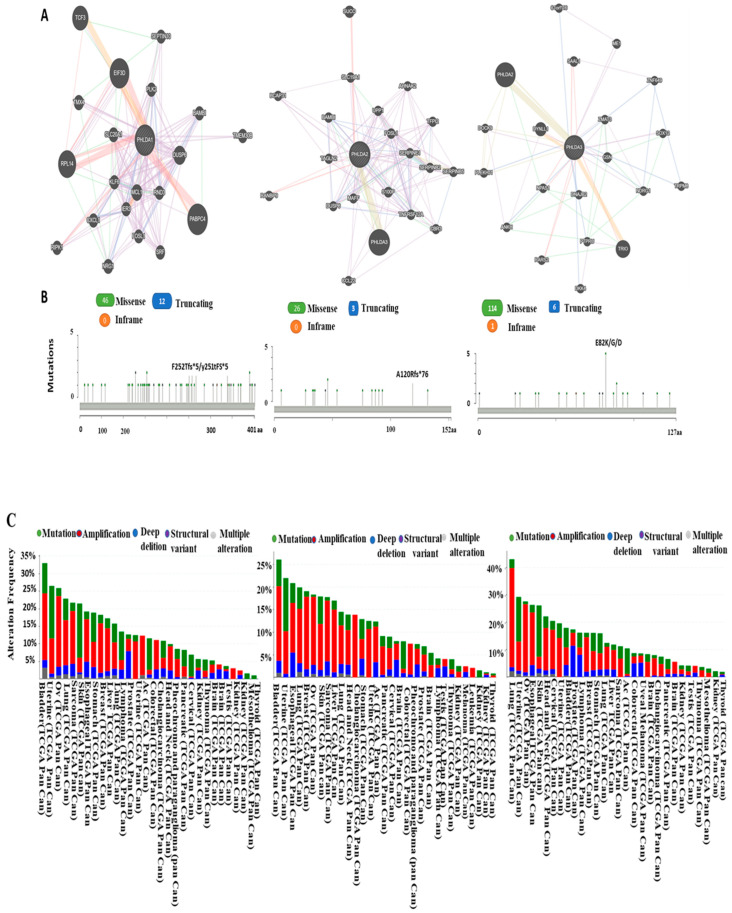

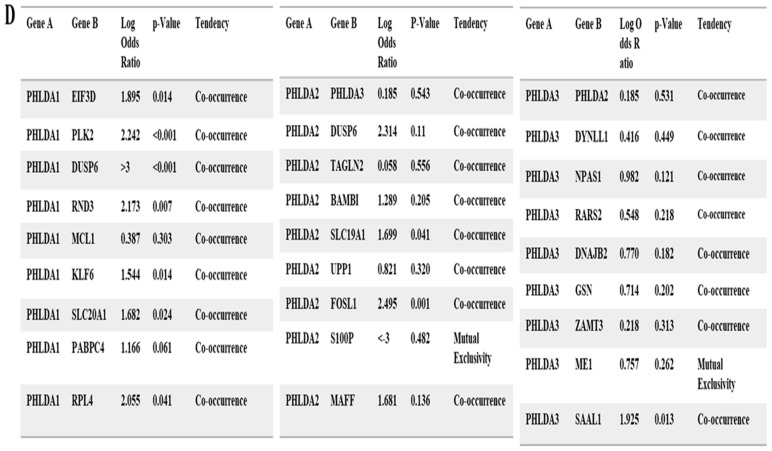
Identification of known and anticipated structural proteins necessary for PHLDA1, PHLDA2, and PHLDA3 function (GeneMANIA), as well as the frequency of mutations and copy number changes (CNAs) in different cancer types (cBioPortal web). (**A**) GeneMANIA displays interacting nodes as circles. After considering co-expression, colocalization, genetic relationships, pathways, physical interactions, and predicted shared protein domains, putative functional partners of PHLDA1, PHLDA2, and PHLDA3 are presented. (**B**) In PHLDA1, 58 mutation sites were identified and were located between amino acids 0 and 401. For PHLDA1, the mutation mainly occurred in colorectal cancer and bladder cancer and existed in a hotspot in the PHLDA domain. For PHLDA2, 29 mutation sites were detected and located between amino acids 0 and 152 of PHLDA2. For Phlda3, 120 mutations were found overall, spanning out between amino acids 0 and 127 of PHLDA3. (**C**). The cBioPortal was used to calculate the frequency of alterations in a ten-gene signature (PHLDA1, EIF3D, PLK2, DUSP6, RND3, MCL1, KLF6, SLC20A1, PABPC4, and RPL14). The alterations included mutations (green), amplifications (red), deep deletions (blue), structural variants (purple), or multiple alterations (grey). With the use of cBioPortal, the alteration frequency of a ten-gene signature (PHLDA2, PHLDA3, DUSP6, TAGLN2, BAMBI, SLC19A1, UPP1, FOSL1, S100P, and MAFF) was determined. The alteration frequency included mutations (green), structural variants (purple), amplifications (red), deep deletions (blue), or multiple alterations (grey). cBioPortal was used to evaluate the alteration frequency of a ten-gene signature (PHLDA3, PHLDA2, DYNLL1, NPAS1, RARS2, DNAJB2, GSN, ZMAT3, ME1, and SAAL1). The alteration frequency included mutations (green), structural variants (purple), amplifications (red), deep deletions (blue), or multiple alterations (grey). (**D**). The co-occurrence of PHLDA1, PHLDA2, and PHLDA3 gene signature alterations and relationships between their respective gene copy number and mRNA expression were discovered via mutual exclusivity panel analysis. The cBioPortal for Cancer Genomics was used to examine the association between PHLDA1, PHLDA2, and PHLDA3 CNAs and mRNA levels.

PPIs are critical cellular events that process downstream signaling and consequently influence cellular activities, such as cell growth and division [44]. Additionally, we used GeneMANIA, which collects information on co-expression, colocalization, genetic interactions, implicated pathways, physical interaction predictions, and shared protein domains to find the PPIs involving these three genes. Here, a protein–protein interaction (PPI) network was used to gain some understanding of the functional interactions of pleckstrin family member A with other closely related proteins at the system level. Along with their respective genes, the predicted protein partners of PHLDA1 are as follows: EIF3D (Eukaryotic Translation Initiation Factor 3 Subunit D), PLK2 (Polo-like kinase 2), DUSP6 (Dual-Specificity Phosphatase 6), RND3 (Rho Family GTPase 3), MCL1 (myeloid cell leukemia-1), KLF6 (KLF Transcription Factor 6), SLC20A1 (Solute Carrier Family 20 Member 1), PABPC4 (Poly(A)-Binding Protein Cytoplasmic 4), and RPL14 (Ribosomal Protein L14) (Figure 3A [PHLDA1]). The following genes and probable protein partners of PHLDA2 are listed as follows: PHLDA3 (pleckstrin 3), DUSP6(Dual-Specificity Phosphatase 6), TAGLN2 (Transgelin 2), BAMBI (BMP and activin membrane-bound inhibitor), SLC19A1 (Solute Carrier Family 19 Member 1), UPP1 (Uridine Phosphorylase 1), FOSL1 (FOS Like 1, AP-1 Transcription Factor Subunit), S100P (S100 Calcium-Binding Protein P), and MAFF (MAF BZIP Transcription Factor F) (Figure 3A [PHLDA2]). The genes for the following predicted functional protein partners of PHLDA3 are as follows: PHLDA2 (pleckstrin 2), DYNLL1 (Dynein Light Chain LC8-Type 1), NPAS1 (Neuronal PAS Domain Protein 1), RARS2 (Arginyl-TRNA Synthetase 2, Mitochondrial), DNAJB2 (DnaJ Heat-Shock Protein Family (Hsp40) Member B2), GSN (Gelsolin), ZMAT3 (Zinc finger matrin-type protein 3), ME1 (Malic Enzyme 1), and SAAL1 (Serum Amyloid A Like 1) (Figure 3A [PHLDA3]). These predicted that pleckstrin interacting partners may have a role in controlling pleckstrin-mediated cancer progression and prognosis.

### 3.4. Analysis of PHLDA Copy Number Alterations and Mutations across Cancers

Using cBioPortal, we evaluated the genetic changes in PHLDA1 in various malignancies and contrasted the findings with those of other important genes listed in the preceding subsection. For PHLDA1, the gene set or pathway was changed in 118 (1%) of the inquired samples, with a somatic mutation frequency of 0.5%. In PHLDA1, 58 mutations were found in patients across numerous samples, as shown in Figure 3B. The mutation sites were situated between amino acids 0 and 401. Among these mutations, 46 missense mutations and 12 truncating variants were found. We also noted that PHLDA1 mutations covered the pleckstrin domain, with a hotspot in F252Tfs*5/Y251Tfs*5, which were primarily seen in colorectal cancer and bladder cancer. The same settings were used to query the PHLDA2 database. In this instance, the somatic mutation rate was 0.2%, and the gene set or pathway was altered in 77 (1%) of the investigated samples. However, compared to PHLDA1, the frequency of somatic mutations decreased. There was a total of 29 mutations in PHLDA2, which were dispersed between amino acids 0 and 152. Therefore, PHLDA2 mutations were thinner than PHLDA1 mutations. Among these mutations, we observed 26 missense mutations, three truncations, and 0 in-frame mutations. The PH domain was similarly spanned by PHLDA2, with hotspots in A120Rfs*76 (Figure 3B). Similar settings as those used for PHLDA2 were also used to query the database for PHLDA3. In this instance, 227 (2%) of the queried samples had altered gene sets or pathways. As a result, the frequency of somatic mutations was 0.2%, which was equivalent to the frequency found for PHLDA2. There were 120 mutations found overall, which were distributed between amino acids 0 and 127. Among these mutations, we observed 114 missense mutations, six truncations, and one in-frame mutation. In bladder cancer, PHLDA3 mutations primarily occurred and spread throughout the plekstrin domain, with an E82K/G/D hotspot (Figure 3B).

Following that, we examined CNAs and mutations using a collection of genes (according to functional protein partners) centered around PHLDA1 (Figure 3A [PHLDA1]). The query was modified and used to pick 10 distinct cancer studies out of 9896 samples, with an alteration frequency of at least 25% (Figure 3C [PHLDA1]; Table S7). The alteration frequency ranged up to 43.07%, as shown in Figure 3C [PHLDA1]. The majority of alterations were found in bladder cancer. Analyzing the mutations and CNAs in the functional partner genes of PHLDA2 revealed alteration frequencies ranging up to 26.11% (Figure 3A [PHLDA2]) (Figure 3C [PHLDA2]; Table S8). Regarding PHLDA3, the query was modified to only select 10 cancer studies out of 9896 samples with an alteration frequency of >30% (Figure 3C [PHLDA3]; Table S9). The alteration frequency was 43.17%, as shown in Figure 3C [PHLDA3]). The list of genes that matched the functional protein partners of PHLDA1 was examined using the co-occurrence analysis sub-tool of cBioPortal, which is based on Fisher’s exact test, to see whether each member was substantially linked. The statistical significance of the co-occurrence of variations in PHLDA1 and each related gene was validated via this analysis (Figure 3D [PHLDA1]). A similar analysis was carried out for PHLDA2 and PHLDA3, demonstrating the statistically significant co-occurrence of changes with PHLDA2 and PHLDA3 as well as mutual exclusivity with its partner genes (Figure 3D [PHLDA2] and [PHLDA3]). As a result, there is abundant evidence that pleckstrin family member A expression may affect cancer development and prognosis by interacting with co-expressed genes and proteins, as shown by the results reported here in combination with those of previous studies.

### 3.5. The Functional Gene Ontology and Pathways of the Genes Associated with the PHLDA Genes

**Figure 4:**
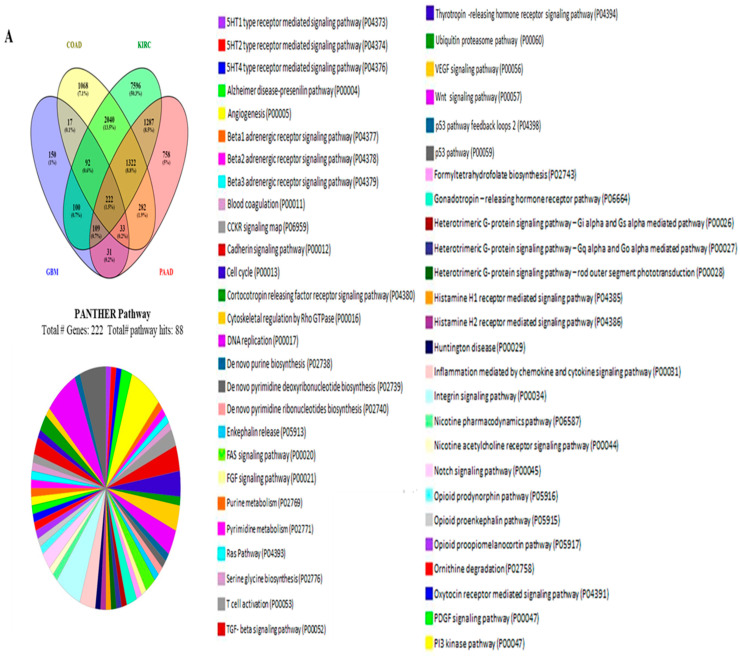

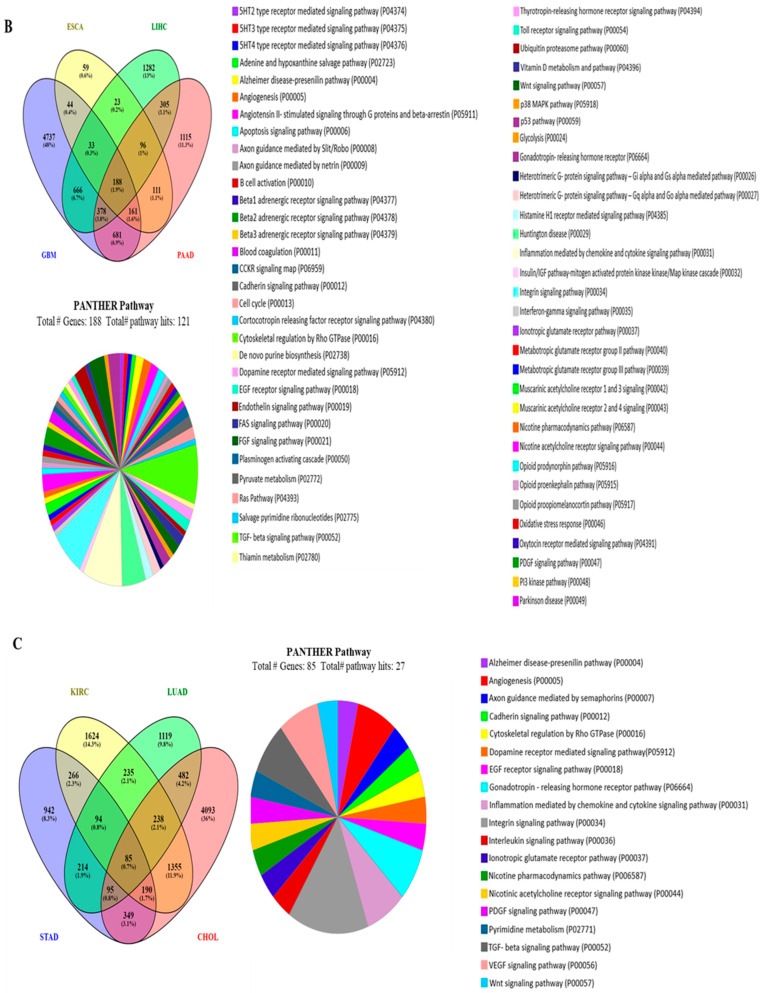
Exploration of the predicted pathways for the genes that were positively correlated with PHLDA1, PHLDA2, and PHLDA3 using PANTHER. (**A**). Venn diagram of the coinciding genes in GBM, COAD, KIRC, and PAAD that are positively correlated with PHLDA1, and PANTHER pathway analysis followed by classification based on the pathways (**B**). ESCA, GBM, LIHC, and PAAD are instances of coincident genes in the Venn diagram of genes that are positively correlated with PHLDA2 and using PANTHER, pathway analysis, and classifications based on those pathways (**C**). Venn diagram of the coinciding genes in STAD, KICH, LUAD, and CHOL that were positively linked with PHLDA3. PANTHER pathway analysis followed by classification based on the pathways.

The normal expression of genes involved in signaling pathways occurs in relation to other genes, and as a result, different genes cumulatively show significant roles in the case of human cancer. We conducted a comprehensive analysis using the R2 platform to detect genes associated with PHLDA1, PHLDA2, and PHLDA3 expression in particular cancers, as described below. Then, separately via Bonferroni correction with a *p*-value < 0.05 for each case, we created a query to find the list of genes that correlated with PHLDA1 in different tumor types. Based on the individual overexpression characteristics of each PHLDA family member, we selected a set of the top four cancer types for each one. We considered each of the four tumor categories for PHLDA1 based on the level of expression: glioblastoma, colon, kidney, and pancreatic cancers. We observed that 754, 5076, 12768, and 4043 genes positively correlated with PHLDA1 expression in glioblastoma (GBM), colon (COAD), ovarian (OV), and pancreatic (PAAD) cancers, respectively. All four cancer types considered in our study matched 222 genes with a positive correlation to PHLDA1 (hereafter referred to as “PHLDA1-correlated gene cluster”) (Figure 4A). In these four tumors, no common genes were found that were negatively correlated with PHLDA1 (Figure S1). For PHLDA2, a cancer set, including glioblastoma, esophageal, liver, and pancreatic cancer, is implemented. In comparison to PHLDA1, fewer genes associated positively with PHLDA2 in each type of cancer, and 188 genes (referred to as the “PHLDA2-correlated gene cluster” above) were common for all four tumors studied (Figure 4B). Additionally, a limited number of 12 genes were found to be negatively linked with PHLDA2 in all cancers investigated (Figure S1). On the other hand, the cancer set for PHLDA3 includes gastric, kidney, lung, and cholangiocarcinoma cancer. In stomach (STAD), kidney (KICH), lung (LUAD), and cholangiocarcinoma (CHOL) tumors, we found that PHLDA3 expression is positively linked with 2236, 4092, 2562, and 6889 genes, respectively. In contrast to PHLDA2, we discovered 85 genes (referred to as the “PHLDA3-centered positive cluster”) that were positively linked with PHLDA3 and were present in every component of cancer set A (Figure 4C). There were no common genes in these four tumors that had a negative correlation with PHLDA3 (Figure S1). According to the above correlation study, PHLDA1, PHLDA2, and PHLDA3 each exhibit distinct correlation connections with other genes in the relevant cancer set of interest. This shows that different gene regulation pathways are common for each PHLDA member and their corresponding associated genes.

According to a recent study, the epithelial–mesenchymal transition (EMT) signaling pathway can be activated by PHLDA family members [11]. The EMT signaling system is a well-known cancer-promoting pathway that activates the potential of tumor cells in various cancers, such as pancreatic cancer, prostate cancer, and breast cancer, to move and -invade. This results in the development of secondary metastatic tumors [45]. As a result, we speculate that the EMT signaling pathway may involve the PH domain of the expression products of the PHLDA family. Then, for the PHLDA1/PHLDA2/PHLDA3-correlated gene clusters, GO and pathway analyses were carried out using the web-based categorization system PANTHER. Each of the relevant genes was subjected to a separate functional classification query, and we reported four key ontologies connected to them. The major molecular functions associated with the PHLDA1-, PHLDA2-, and PHLDA3-correlated gene clusters are shown in Figures S2–S4. Our findings indicated that while PHLDA1, PHLDA2, and PHLDA3 are involved in regulating various signaling pathways, depending on the tissue, they exhibit comparable correlations in other signaling pathways. We looked at how the linked genes were classified functionally for each GO, allowing for a corrected *p*-value of less than 0.05. Each of the correlated genes was subjected to a distinct functional classification query, and the four key ontologies related to them were provided. Multiple sub-ontologies made up each of the ontologies. Our study showed that the primary biological processes connected to the PHLDA1-correlated gene cluster were biological control, cellular processes, and metabolic processes (Figure S2). On the other hand, the main biological processes of the PHLDA2-correlated gene cluster were biological regulation, cellular processes, and metabolic processes (Figure S3). Additionally, the PHLDA3-correlated gene cluster was mostly involved in biological regulation, cellular processes, and signaling, which are their major biological processes (Figure S4). Additionally, “Binding” was the main biological process associated with the PHLDA1, PHLDA2, and PHLDA3-correlated gene clusters (Figures S2–S4). The main cellular components associated with the PHLDA1-, PHLDA2-, and PHLDA3-correlated gene clusters were “Cellular anatomical entity” and “Protein-containing complex” (Figures S2–S4). The PHLDA1-correlated gene cluster affected 88 pathways; the PHLDA2-correlated gene cluster affected 121 pathways; and the PHLDA3-correlated gene cluster affected 27 pathways, with a wider range of roles (Figure 4A–C). The most significant pathway associated with the PHLDA1-correlated gene cluster was the 5HT1-type receptor-mediated signaling pathway. The de novo purine biosynthesis pathway was the major player in the PHLDA2-correlated gene cluster, whereas the integrin signaling pathway was the most significant pathway associated with the PHLDA3-correlated gene cluster. However, twelve common pathway classes occurred in these three gene clusters, including those involved in 5-hydroxytryptamine Alzheimer disease-presenilin, Angiogenesis, Cadherin signaling, Gonadotropin-releasing hormone receptor, Cytoskeletal regulation by Rho GTPase, cytokine, and Inflammation mediated by chemokine, Integrin, Nicotine pharmacodynamics, Nicotine acetylcholine receptor, PDGF signaling, TGF-beta signaling, and Wnt signaling. In conclusion, our findings indicated that PHLDA1, PHLDA2 and PHLDA3 exhibit comparable correlations in specific signaling pathways, depending on the tissue; furthermore, they participate in the regulation of different signaling pathways.

## 4. Discussion

PHLDA family members are considered to be potential targets for cancer treatments as they are expressed in various types of cancer [11]. However, their complete expression patterns, prognostic significance, putative functions, and drug interaction networks in various cancer types remain largely unclear. We used comparative data mining to analyze a variety of gene expression datasets to deliver a systematic understanding of the function of the PHLDA gene family in cancer diagnosis [46]. In addition, according to Oncomine-based expression analysis, PHLDA1 was found to be upregulated in brain, colorectal, kidney, lymphoma, melanoma, myeloma, ovarian, and pancreatic cancer but downregulated in breast, esophageal, cervical, liver cancer, head and neck, and lymphoma cancer. PHLDA2 was elevated in a variety of cancers; however, it was downregulated in sarcoma cancer. These cancers included those of the brain, breast, colorectal, esophageal, head and neck, kidney, lung, and lymphoma. In contrast, PHLDA3 was upregulated in colorectal, gastric, kidney, and lymphoma cancer but downregulated in esophageal and sarcoma cancer. The obtained patterns of gene expression indicate that PHLDA1, PHLDA2, and PHLDA3 are differentially expressed in cancerous cells compared to normal tissues and that the degree of their expression also changes depending on tissue type.

Further we investigated the link between pleckstrins’ expression levels and OS in various cancers to better understand the value of pleckstrins as prognostic markers. The prognostic value was evaluated using PrognoScan, R2, and Kaplan–Meier Plotter. In general, brain, ovarian, cholangiocarcinoma, and uterine cancers were associated with high PHLDA1 expression and poor prognoses. Its role in breast cancer was not clear. Our findings are in line with those of several earlier pieces of research. For example, colon cancer has been associated with PHLDA1 overexpression, and PHLDA1 knockdown prevents anchorage-independent cell growth and reduces cell migration in colon cancer cells [16]. Reduced PHLDA1 expression promotes the development of breast cancer and may be an effective prognostic indicator of prognosis. The downregulation of PHLDA1 and estrogen receptor expression in breast cancers have also been linked [47]. Furthermore, in estrogen-receptor-positive breast cancers, PHLDA1 mRNA expression is high and linked to a high risk of distant metastasis. This suggests that PHLDA1, depending on the estrogen receptor status, which affects the clinical behavior of tumors, may play various roles in breast cancer cells. PHLDA1, which also occurs in all normal gastric mucosa, is a component of a primordial stem-like cell signature in normal human intestinal epithelial crypt cells [8]. Next, we observed that the upregulation of PHLDA2 was associated with poor OS in esophageal, gastric, liver, ovarian, uterine, and cholangiocarcinoma cancer. Our results agree with some previous studies. In gastric cancer, the HGF (hepatocyte growth factor)-mediated overexpression of PHLDA2 is linked to apoptosis [32]. However, their role in colon cancer indicates contradictory results. It shows both high and low expression in different CRC research studies (Figure 2B and Figure 4B). Therefore, further study is needed to draw conclusions on PHLDA2 gene expression in CRC studies. Moreover, any significant variation (high or low) in PHLDA2 expression can be used as a reliable predictive indicator of poor prognosis in ovarian cancer patients. Because the PHLDA3 gene is overexpressed in cancers of the stomach, lung, sarcoma, and uterus, PHLDA3 can also encourage the proliferation of lung cancer cells and invasion by turning on the Wnt signaling pathway, according to Lei et al. [48]. Lower expression was observed in bladder, brain, breast, colon, head and neck squamous, and liver cancers. It has been observed that the nuclear accumulation of p53, an indicator of harmful TP53 mutations, has been associated with low or absent PHLDA3 expression in lobular breast cancer [49]. According to a recent study, the negative feedback regulators of the PI3K pathway act as tumor suppressors and PHLDA3 is a negative feedback regulator of the PI3K pathway of head and neck squamous cell carcinoma (HNSC). For the diagnosis of patients with HNSC, PHLDA3 expression levels could deliver promising results [50]. Based on mRNA expression and clinical data from Oncomine and TCGA, we infer that PHLDA1 has an oncogenic role in the case of brain, colon, lymphoma, kidney, and pancreatic cancers. PHLDA2 has an oncogenic role in brain, colon, esophageal, ovarian, lung, and pancreatic cancers. On the other hand, PHLDA3 is observed in brain, lymphoma, kidney, and cholangiocarcinoma cancers.

Cancer is a multi-step process in which malignant cells develop over time as a result of a series of progressive mutations. Four main variables, including epigenetic and somatically acquired genomic, proteomic, and transcriptome changes, affect tumorigenesis [44]. Somatic cells can develop acquired or somatic mutations on their own, leading to cellular transformation. Certain gene alterations have an impact on a variety of malignancies. Numerous cancer types frequently exhibit CNAs and somatic abnormalities to the chromosomal structure that result in the addition or loss of copies of certain DNA regions [51]. To identify human cancers with CNAs and pleckstrin gene alterations, we employed cBioPortal. The majority of missense and truncating mutations originated in protein-coding regions. It has been established that the E17K mutation in the AKT1 protein’s pleckstrin homology (PH) domain encourages protein phosphorylation and membrane localization, which, in turn, results in cancer [52]. The distinct split P^H^ domain of PHLDA1 is interrupted by a polyglutamine region (PolyQ). The genomic instability hotspots identified as PolyQ regions can change the stability of the translated proteins by expanding or contracting during replication [32]. PHLDA2 expression is found to be induced by oncogenic EGFR/AKT signaling in lung cancers via the correlation of PHLDA2 expression with both p-AKT and EGFR mutations [53]. The function of PHLDA2 in cancers is still largely unclear. According to Rieko et al., the genomic region of the PHLDA3 gene undergoes loss of heterozygosity (LOH) at high frequency in human PanNETs; furthermore, the PHLDA3 promoter is methylated [54]. Here, we discovered many missense and truncating mutations in the PHLDA family gene protein-coding regions in various cancer types through our systematic analysis. Experimental confirmation of these findings is still required. PHLDA1 mutations mainly occur in colorectal cancer and bladder cancer and consist of several frame-shift insertions or deletion mutations in a hotspot at position p. F252Tfs*5/Y251Tfs*5 in the pleckstrin domain. The pleckstrin domain was similarly spanned by PHLDA2, with hotspots at position p. A120Rfs*76. Specifically, PHLDA3 mutations mainly in bladder cancer and spread throughout the pleckstrin domain, consisting of several frame-shift insertions or deletion mutations in a hotspot at position p. E82K/G/D. Although this has not yet been confirmed, these mutations may play a part in the control of cancer progression and prognosis.

PPIs (protein–protein interactions) are crucial in many biological processes. A variety of diseases, including cancer, infectious disorders, and neurodegenerative diseases, are linked to abnormal PPIs [55]. As a result, we initially used GeneMANIA to construct the interaction network of the top 10 highly associated functional protein partners of PHLDA1, PHLDA2, and PHLDA3. The use of cBioPortal revealed that genetic modifications of the ten genes in the PHLDA1, PHLDA2, and PHLDA3 signatures primarily occurred in bladder and lung cancers, with alteration frequencies ranging between 35.54%, 26.11%, and 43.17%, respectively. The genomic alterations appeared to affect the expression of metastatic and EMT genes, and activity scores correlated positively with other cancer-relevant pathways [56]. Therefore, in order of increasing pathway activity scores, the PHLDA family plays a dominant role.

The members of the PHLDA family might have oncogenic functions and might be used as novel biomarkers. Since these pleckstrins are highly expressed in some cancers, the R2 genomics platform was utilized to find genes associated with PHLDA1, PHLDA2, and PHLDA3. Here, it was found that PHLDA1 is highly correlated with genes found in brain, colon, kidney, and pancreatic cancers. Here, 222 genes with a positive correlation with PHLDA1 were found. Brain, esophagus, liver, and pancreatic cancers were positively associated with PHLDA2 genes, and 188 of these genes were common in all cancers. For PHLDA3, positively correlated genes were detected in gastric, kidney, lung, and cholangiocarcinoma cancer, respectively, and 85 genes were found to be common in all cancers. Next, we utilized PANTHER to carry out GO and pathway analysis to ascertain the common role of these associated genes in the aforementioned cancers. We observed that 88,121,27 pathways were impacted by PHLDA1-centered positive, PHLDA2-centered positive, and PHLDA3-centered positive clusters, respectively. Only 12 pathways were common between PHLDA1, PHLDA2, and PHLDA3. Our correlation study revealed that PHLDA1, PHLDA2, and PHLDA3 have separate roles in terms of pathway regulation. Therefore, they could perform a few of the identical roles found in certain signaling pathways and particular cancer types.

The development of new biomarkers and therapeutic targets, as well as a better understanding of the molecular mechanism underlying carcinogenesis, significantly depends on the integration of multiomic data [57]. The above systematic data of PHLDA gene family members were obtained from publicly available clinical data. These findings imply that PHLDA gene expression may be applied in clinical settings and that the co-expression of pleckstrin may influence clinical outcomes in patients with certain cancers. Additionally, we showed that PHLDA1, PHLDA2, and PHLDA3 have distinct roles in pathway regulation.

## 5. Conclusions

In this study, we thoroughly investigated the expression, mutations, CNAs, functional protein partners, related genes, and prognostic value of the PHLDA gene family in a variety of human malignancies using a range of online bioinformatics platforms and methods. PHLDA1, 2, and 3 are all AKT inhibitors. Additionally, the PHLDA family members have been increasingly understood to be promising targets for cancer therapies due to the activation of PHLDA genes in a variety of cancer types. However, their detailed expression patterns, prognostic significance, and possible roles are still completely unknown. Our multiomics research showed that PHLDA1, PHLDA2, and PHLDA3 have various functions in the development of cancer and regulate the prognosis of patient’s cancer differently. The co-expression of PHLDA1, PHLDA2, and PHLDA3, as well as their connection to the EMT signaling pathway, and the expression of the p53 protein, may help to predict patient outcomes and responses to target therapy. Among the PHLDA family, PHLDA1 might serve as a therapeutic target for several cancers, such as colon, ovarian, and pancreatic cancer, while PHLDA2 might be used as a therapeutic target for esophageal, kidney, and ovarian cancer. It may be possible to use PHLDA3 as a therapeutic target for colon, gastric, and kidney cancer. Pleckstrin, PPI, and co-expression analysis may also be used to predict the probable underlying signaling processes related to the role of PHLDA family members in particular cancers.

Nevertheless, there are certain limitations to this study. At the outset, our research comprised analyses based on previous data; hence, experimental data are still required to validate our findings. To support our findings, we recommend carrying out additional investigations.

However, this study is predicted to further our knowledge of the molecular and clinical prognoses of many types of cancer. It also offers new perspectives on the molecular processes underlying cancer, which will aid in translating genetic information into therapeutic applications. Nevertheless, as data mining-based analyses may manifest result in overfitting and underfitting, further theoretical, experimental, and clinical research is needed to corroborate the findings of this study.

## Data Availability

All of the data generated or analyzed during this study are included in this published article (and its Supplementary Information Files).

## Supplementary Materials

The following supporting information can be downloaded at: https://www.mdpi.com/article/10.3390/cimb46060328/s1, Supplementary Table S1. PHLDA1 expression in various cancers from the Oncomine database. Supplementary Table S2. PHLDA2 expression in various cancers from the Oncomine database. Supplementary Table S3. PHLDA3 expression in various cancers from the Oncomine database. Supplementary Table S4. Association of PHLDA1 expression and survival of cancer patients (PrognoScan database). Supplementary Table S5. Association of PHLDA2 expression and survival of cancer patients (PrognoScan database). Supplementary Table S6. Association of PHLDA3 expression and survival of cancer patients (PrognoScan database). Supplementary Table S7. Alteration frequency of a ten-gene signature (PHLDA1, EIF3D, PLK2, DUSP6, RND3, MCL1, KLF6,SLC20A1, PABPC4, and RPL14) in various cancers (cBioPortal web). Supplementary Table S8. Alteration frequency of a ten-gene signature (PHLDA2, PHLDA3, DUSP6, TAGLN2, BAMBI, SLC19A1, UPP1, FOSL1, S100P, and MAFF) in various cancer (cBioPortal web). Supplementary Table S9. Alteration frequency of a ten-gene signature (PHLDA3, PHLDA2, DYNLL1, NPAS1, RARS2, DNAJB2, GSN, ZMAT3, ME1, and SAAL1) in various cancer (cBioPortal web). Supplementary Figure S1. Venn diagram shows the coincident genes with PHLDA1 (i), PHLDA2 (ii), and PHLDA3 (iii) that are negatively associated. Supplementary Figure S2. Gene ontology (GO) analysis of positively correlated genes of *PHLDA1* using PANTHER. Here, Molecular function (i), Biological Process (ii), and Cellular component (iii). Supplementary Figure S3. Gene ontology (GO) analysis of positively correlated genes of *PHLDA2* using PANTHER. Here, Molecular function (i), Biological Process (ii), and Cellular component (iii). Supplementary Figure S4. Gene ontology (GO) analysis of positively correlated genes of *PHLDA3* using PANTHER. Here, Molecular function (i), Biological Process (ii), and Cellular component (iii). Supplementary Figure S5. Gene ontology (GO) analysis of positively correlated genes of *PHLDA3* using PANTHER. Here, Molecular function (i), Biological Process (ii), and Cellular component (iii).
