# Supplementary material for: Multiomics Analysis of the PHLDA Gene Family in Different Cancers and Their Clinical Prognostic Value
Source: Curr Issues Mol Biol. 2024 May 30;46(6):5488–510. doi: 10.3390/cimb46060328 (PMC11201736; doi:10.3390/cimb46060328)
Supplement: Supplementary file 1 [file cimb-46-00328-s001.zip › cimb-2988307-supplemnetary.pdf]

Supplementary Tables

Supplementary Table S1. PHLDA1 expression in various cancers from the Oncomine database.

Cancer	Cancer subtype	p-value	Fold change	Rank (%)	Sample	Reference
Brain	Oligodendroglioma	2.55E-17	3.416	1	180	(Sun et al., 2006)
	Glioblastoma	8.30E-20	2.903	1	180	(Sun et al., 2006)
	Anaplastic Astrocytoma	3.63E-7	2.461	3	180	(Sun et al., 2006)
	Anaplastic Oligoastrocytoma	9.51E-6	6.347	1	33	(French et al., 2005)
	Anaplastic Oligodendroglioma	2.23E-7	5.73	2	33	(French et al., 2005)
	Glioblastoma	2.64E-9	3.511	2	54	(Bredel et al., 2005)
	Brain Glioblastoma	4.72E-10	3.550	4	557	TCGA
	Glioblastoma	1.72E-8	2.377	3	84	(Murat et al., 2008)
Breast	Invasive Ductal Breast Carcinoma	5.54E-7	-16.882	1	66	(Ma et al., 2009)
	Ductal Breast Carcinoma in Situ	1.84E-6	-10.087	1	66	(Ma et al., 2009)
	Invasive Lobular Breast Carcinoma	9.38E-9	-2.537	9	593	TCGA
Cervical	High Grade Cervical Squamous Intraepithelial Neoplasia	9.53E-5	-3.731	2	41	(Ma et al., 2009)
	Cervical Squamous Cell Carcinoma	1.86E-5	-2.157	6	66	(Scotto et al., 2008)
Colorectal	Colorectal Carcinoma	3.29E-20	6.686	1	105	(Skrzypczak et al., 2010)
	Colorectal Adenocarcinoma	1.24E-21	6.684	1	105	(Skrzypczak et al., 2010)

Colorectal Adenoma	5.86E-7	2.342	1	78	(Gaspar et al., 2008)
Colon Adenoma	2.23E-9	8.212	1	40	(Skrzypczak et al., 2010)
Colon Adenoma	5.52E-8	3.534	2	40	(Skrzypczak et al., 2010)
Colon Carcinoma	1.10E-8	6.033	3	40	(Skrzypczak et al., 2010)
Colon Carcinoma	6.81E-7	2.651	8	40	(Skrzypczak et al., 2010)
Colon Mucinous Adenocarcinoma	4.62E-14	8.109	1	237	TCGA
Rectal Mucinous Adenocarcinoma	1.08E-6	11.495	2	237	TCGA
Rectosigmoid Adenocarcinoma	5.01E-7	2.591	2	237	TCGA
Cecum Adenocarcinoma	1.04E-11	5.851	3	237	TCGA
Rectal Adenocarcinoma	1.81E-13	6.095	7	237	TCGA
Rectal Adenocarcinoma	6.78E-32	3.898	1	130	(Gaedcke et al., 2010)
Rectal Adenocarcinoma	1.15E-5	3.130	2	105	(Kaiser et al., 2007)
Colon Mucinous Adenocarcinoma	1.77E-6	3.536	3	105	(Kaiser et al., 2007)
Rectosigmoid Adenocarcinoma	4.90E-5	2.806	6	105	(Kaiser et al., 2007)
Cecum Adenocarcinoma	3.10E-5	2.619	8	105	(Kaiser et al., 2007)
Colon Adenoma	2.33E-14	4.933	2	64	(Sabates-Bellver et al., 2007)
Rectal Adenoma	2.94E-5	7.588	8	64	(Sabates-Bellver et al., 2007)

	Colorectal Carcinoma	1.66E-12	7.220	3	82	(Hong et al., 2010)
Esophagus	Barrett's Esophagus	4.41E-7	-4.319	2	52	(Wang et al., 2006)
	Esophageal Adenocarcinoma	5.26E-5	-6.831	5	52	(Wang et al., 2006)
	Esophageal Adenocarcinoma	8.77E-24	-8.856	2	118	(Kim et al., 2010)
	Barrett's Esophagus	1.75E-8	-16.920	9	118	(Kim et al., 2010)
	Esophageal Squamous Cell Carcinoma	2.02E-8	-4.170	4	34	(Hu et al., 2010)
	Esophageal Squamous Cell Carcinoma	1.23E-12	-2.471	3	106	(Su et al., 2011)
Kidney	Clear Cell Renal Cell Carcinoma	1.35E-5	2.186	3	44	(Higgins et al., 2003)
Liver	Hepatocellular Carcinoma	1.54E-25	-4.366	1	197	(Chen et al., 2002)
	Hepatocellular Carcinoma	1.10E-6	-2.339	2	75	(Wurmbach et al., 2007)
Lymphoma	Germinal Center B-Cell-Like Diffuse Large B-Cell Lymphoma	2.11E-9	2.652	1	136	(Compagno et al., 2009) Compagno
	Follicular Lymphoma	1.56E-21	2.857	2	136	(Compagno et al., 2009)
	Diffuse Large B-Cell Lymphoma	2.53E-19	2.989	3		(Compagno et al., 2009)
	Diffuse Large B-Cell Lymphoma	2.53E-19	2.989	3	136	(Compagno et al., 2009)
	Activated B-Cell-Like Diffuse Large B-Cell Lymphoma	5.79E-7	2.911	10	136	(Compagno et al., 2009)
	Unspecified Peripheral T-Cell Lymphoma	1.28E-13	-6.514	2	60	(Piccaluga et al., 2007)

	Angioimmunoblastic T-Cell Lymphoma	2.37E-5	-6.341	6	60	(Piccaluga et al., 2007)
Melanoma	Melanoma	2.27E-5	11.079	1	37	(Haqq et al., 2005)
	Cutaneous Melanoma	3.99E-9	4.490	2	70	(Talantov et al., 2005)
	Benign Melanocytic Skin Nevus	6.39E-5	9.571	4	70	(Talantov et al., 2005)(24)-Dmitri Talantov
Myeloma	Multiple Myeloma	9.38E-14	2.599	1	158	(Agnelli et al., 2009)
Ovarian	Ovarian Carcinoma	1.05E-7	2.012	10	195	(Bonome et al., 2008)
Pancreas	Pancreatic Carcinoma	1.65E-8	2.578	2	52	(Pei et al., 2009)
	Pancreatic Ductal Adenocarcinoma	1.39E-8	2.501	8	58	(Badea et al., 2008)

Supplementary Table S2. PHLDA2 expression in various cancers from the Oncomine database.

**Cancer**	**Cancer subtype**	**p-value**	**Fold change**	**Rank (%)**	**Sample**	**Reference**
Brain	Glioblastoma	2.08E-6	8.475	3	101	(Lee et al., 2006)
Breast	Ductal Breast Carcinoma in Situ	6.61E-5	3.366	2	66	(Ma et al., 2009)
Colorectal	Colon Mucinous Adenocarcinoma	6.01E-9	2.974	6	237	TCGA
	Colorectal Carcinoma	6.96E-7	2.116	7	105	(Skrzypczak et al., 2010)
	Colorectal Carcinoma	1.85E-8	2.701	8	82	(Hong et al., 2010)
Esophagus	Barrett's Esophagus	1.235E-12	3.064	1	118	(Kim et al., 2010)

	Barrett's Esophagus	6.99E-5	2.532	5	52	(Wang et al., 2006)
Head and Neck	Tongue Squamous Cell Carcinoma	7.53E-12	5.155	2	93	(Talbot et al., 2005)
	Tongue Squamous Cell Carcinoma	8.55E-11	6.263	2	58	(Estilo et al., 2009)
Kidney	Clear Cell Renal Cell Carcinoma	2.30E-5	4.012	4	67	(Yusenko et al., 2009)
	Renal Pelvis Urothelial Carcinoma	5.25E-6	3.263	8	92	(Jones et al., 2005)
Lung	Lung Adenocarcinoma	2.05E-11	2.799	1	96	(Beer et al., 2002)
	Lung Adenocarcinoma	1.99E-19	4.027	1	107	(Landi et al., 2008)
	Lung Adenocarcinoma	9.11E-8	4.015	2	39	(Stearman et al., 2005)
	Lung Adenocarcinoma	1.09E-7	3.634	2	66	(Su et al., 2007)
	Lung Adenocarcinoma	1.42E-17	2.387	2	116	(Selamat et al., 2012)
	Squamous Cell Lung Carcinoma	1.18E-6	2.744	10	93	(Talbot et al., 2005)
Lymphoma	Diffuse Large B-Cell Lymphoma	1.12E-27	5.683	1	136	(Piccaluga et al., 2007)
	Activated B-Cell-Like Diffuse Large B-Cell Lymphoma	1.01E-11	4.274	2	136	(Piccaluga et al., 2007)
	Follicular Lymphoma	1.18E-18	2.958	3	136	(Piccaluga et al., 2007)
	Germinal Center B-Cell-Like Diffuse Large B-Cell Lymphoma	1.18E-18	3.675	5	136	(Piccaluga et al., 2007)
	Hodgkin's Lymphoma	1.35E-7	6.336	1	67	(Brune et al., 2008)

	Centroblastic Lymphoma	1.25E-9	4.164	6	336	(Basso et al., 2005)
	Diffuse Large B-Cell Lymphoma	1.01E-5	2.591	6	336	(Basso et al., 2005)
Ovarian	Ovarian Mucinous Adenocarcinoma	8.87E-5	3.756	1	50	(Lu et al., 2004)
	Ovarian Mucinous Adenocarcinoma	8.87E-9	2.178	1	103	(Hendrix et al., 2006)
Pancreas	Pancreatic Ductal Adenocarcinoma	6.53E-14	7.481	1	78	(Badea et al., 2008)
	Pancreatic Carcinoma	8.27E-7	9.600	3	52	(Pei et al., 2009)

Supplementary Table S3. *PHLDA3* expression in various cancers from the Oncomine database.

**Cancer**	**Cancer subtype**	**p-value**	**Fold change**	**Rank (%)**	**Sample**	**Reference**
Colorectal	Colon Adenocarcinoma	4.23E-13	3.297	3	123	(Ki et al., 2007)
Esophagus	Esophageal Adenocarcinoma	1.47E-19	-3.107	3	118	(Kim et al., 2010)
	Barrett's Esophagus	8.64E-9	-3.315	8	118	(Kim et al., 2010)
Gastric	Gastric Intestinal Type Adenocarcinoma	6.63E-7	2.481	1	90	(Cho et al., 2011)
Kidney	Clear Cell Renal Cell Carcinoma	2.31E-9	2.190	9	92	(Jones et al., 2005)
Lymphoma	Diffuse Large B-Cell Lymphoma	1.56E-22	2.188	2	136	(Compagno et al., 2009)
	Germinal Center B-Cell-Like Diffuse Large B-Cell Lymphoma	4.68E-7	2.470	2	136	(Compagno et al., 2009)
	Activated B-Cell-Like Diffuse Large B-Cell Lymphoma	7.67E-9	2.104	5	136	(Compagno et al., 2009)

Other	Testicular Seminoma	5.18E-13	5.144	1	74	(Sperger et al., 2003)
	Mixed Germ Cell Tumor, NOS	9.65E-15	2.211	1	107	(Korkola et al., 2006)
	Seminoma, NOS	2.02E-8	5.102	3	107	(Korkola et al., 2006)
	Seminoma, NOS	8.74E-6	2.434	4	107	(Korkola et al., 2006)
	Embryonal Carcinoma, NOS	4.43E-7	2.412	7	107	(Korkola et al., 2006)
Sarcoma	Pleomorphic Liposarcoma	8.98E-7	-2.378	3	158	(Barretina et al., 2010)
	Pleomorphic Myxofibrosarcoma	9.29E-5	-2.070	3	158	(Barretina et al., 2010)
	Myxoid/Round Cell Liposarcoma	4.43E-7	-2.812	4	158	(Barretina et al., 2010)
	Myxofibrosarcoma	2.69E-6	-2.248	6	158	(Barretina et al., 2010)
	Leiomyosarcoma	8.91E-6	-2.138	6	158	(Barretina et al., 2010)
	Dedifferentiated Liposarcoma	2.06E-5	-2.013	7	158	(Barretina et al., 2010)

Supplementary Table S4. Association of PHLDA1 expression and survival of cancer patients (PrognoScan database).

Cancer type	Endpoint	N	Dataset	Probe ID	Cox P-value	HR [95% CI^{low}- CI^{upp}]
Bladder cancer	Overall survival	30	GSE5287	218000_s_at	0.0036	2.78 [1.40 - 5.51]
Brain cancer	Overall survival	70	GSE7696	217997_at	0.0083	0.73 [0.57 - 0.92]

Brain cancer	Overall survival	70	GSE7696	217997_at	0.0324	0.77[0.06-0.98]
Brain cancer	Overall survival	70	GSE7696	217996_at	0.0175	0.69[0.51-0.94]
Brain cancer	Overall survival	70	GSE7696	217998_at	0.0056	0.36[0.17-0.74]
Brain cancer	Overall survival	50	MGH-glioma	35909_at	0.0197	0.73[0.56-0.95]
Brain cancer	Overall survival	74	GSE4412-GPL96	217997_at	0.0008	0.48[0.31-0.74]
Brain cancer	Overall survival	74	GSE4412-GPL96	218000_s _at	0.0259	0.76[0.60-0.97]
Brain cancer	Overall survival	74	GSE4412-GPL96	217998_at	0.0033	0.55[0.37-0.82]
Brain cancer	Overall survival	74	GSE4412-GPL96	217996_at	0.0065	0.55[0.36-0.85]
Brain cancer	Overall survival	74	GSE4412-GPL96	217999_s _at	0.0001	0.45[0.28-0.73]
Brain cancer	Overall survival	74	GSE4412-GPL97	225842_at	0.2191	0.59[0.38-0.93]
Breast cancer	Relapse-free survival	60	GSE1397	4920	0.0018	0.17[0.06-0.52]
Breast cancer	Overall survival	155	GSE9893	6574	0.0042	0.49[0.30-0.80]
Breast cancer	Distant metastasis-free survival	286	GSE2034	218000_s _at	0.0054	0.78[0.66-0.93]
Breast cancer	Disease-specific survival	159	GSE1456-GPL96	218000_s _at	0.0388	1.44[1.02-2.03]
Breast cancer	Disease-specific survival	159	GSE1456-GPL96	217996_at	0.0358	1.79[1.04-3.09]

Breast cancer	Distant metastasis-free survival	117	E-TABM-158	217998_at	0.0468	0.38[0.14-0.99]
Breast cancer	Relapse-free survival	198	GSE7390	217996_at	0.0297	1.20[1.02-1.43]
Breast cancer	Relapse-free survival	198	GSE7390	217997_at	0.0359	1.20[1.01-1.43]
Colorectal cancer	Disease free survival	145	GSE17536	217996_at	0.0300	1.59[1.05-2.42]
Colorectal cancer	Disease free survival	145	GSE17536	218000_s _at	0.0482	2.00[1.01-3.99]
Colorectal cancer	Disease Specific survival	177	GSE17536	217996_at	0.0317	1.45[1.03-2.02]
Colorectal cancer	Overall survival	177	GSE17536	217996_at	0.0419	1.35[1.01-1.80]
Colorectal cancer	Disease free survival	226	GSE14333	217997_at	0.0299	1.42[1.03-1.96]
Colorectal cancer	Disease free survival	226	GSE14333	217996_at	0.0131	1.52[1.09-2.13]
Colorectal cancer	Disease free survival	55	GSE17537	217999_s_at	0.0288	1.75[1.06-2.89]
Colorectal cancer	Overall survival	55	GSE17537	217997_at	0.0406	1.62[1.02-2.58]
Colorectal cancer	Disease free survival	55	GSE17537	217997_at	0.0283	1.82[1.07-3.11]
Colorectal cancer	Overall survival	55	GSE17537	217996_at	0.0222	1.76[1.08-2.86]
Colorectal cancer	Disease Specific	49	GSE17537	217997_at	0.0127	2.35[1.20-4.61]

	survival					
Colorectal cancer	Disease free survival	55	GSE17537	217996_at	0.0096	2.15[1.20-3.83]
Colorectal cancer	Disease Specific survival	49	GSE17537	217996_at	0.0165	2.28[1.16-4.47]
Esophagus cancer	Overall survival	34	GSE11595	667883	0.00578	1.80[1.18-2.72]
Eye cancer	Distant metastasis-free survival	63	GSE22138	217998_at	0.0027	0.42[0.24-0.74]
Eye cancer	Distant metastasis-free survival	63	GSE22138	217997_at	0.0002	0.70[0.57-0.84]
Eye cancer	Distant metastasis-free survival	63	GSE22138	225842_at	0.0028	0.66[0.50-0.87]
Eye cancer	Distant metastasis-free survival	63	GSE22138	217996_at	0.0001	0.66[0.54-0.81]
Eye cancer	Distant metastasis-free survival	63	GSE22138	217999_s_at	0.0001	0.57[0.41-0.79]
Lung cancer	Overall survival	82	Jacob-00182-CANDF	217996_at	0.0293	1.63[1.05-2.53]
Lung cancer	Overall survival	82	Jacob-00182-CANDF	217997_at	0.0309	1.70[1.05-2.74]
Lung cancer	Overall survival	82	Jacob-00182-CANDF	218000_s _at	0.0054	1.71[1.17-2.50]
Lung cancer	Relapse-free survival	204	GSE31210	217998_at	0.0221	0.57[0.35-0.92]
Lung cancer	Overall survival	111	GSE3141	217996_at	0.0084	1.53[1.11-2.10]

Lung cancer	Overall survival	111	GSE3141	225842_at	0.0389	1.56[1.02-2.37]
Lung cancer	Overall survival	111	GSE3141	217997_at	0.0314	1.55[1.04-2.32]
Lung cancer	Overall survival	50	GSE4716-GPL3694	2733	0.0393	1.94[1.03-3.65]
Ovarian cancer	Overall survival	133	DUKE-OC	217996_at	0.0309	0.86[0.76-0.99]
Ovarian cancer	Overall survival	133	DUKE-OC	217997_at	0.0395	.85[0.72-0.99]
Ovarian cancer	Disease free survival	185	GSE26712	217998_at	0.0352	1.49[1.03-2.17]
Ovarian cancer	Progression free survival	110	GSE17260	A_24_P943597	0.0331	0.82[0.69-0.99]
Skin cancer	Overall survival	38	GSE19234	218000_s _at	0.0056	1.87[1.20-2.91]
Skin cancer	Overall survival	38	GSE19234	217999_s_at	0.0261	2.28[1.10-4.72]
Skin cancer	Overall survival	38	GSE19234	217998_at	0.0315	2.50[1.08-5.78]
Skin cancer	Overall survival	38	GSE19234	217997_at	0.0172	2.09[1.14-3.82]

Supplementary Table 5. Association of PHLDA2 expression and survival of cancer patients (PrognoScan database).

**Cancer type**	**Endpoint**	**N**	**Dataset**	**Probe ID**	**Cox P-value**	**HR [95% CI^{low}- CI^{upp}]**
Bladder cancer	Disease Specific survival	165	GSE 13507	ILMN_1671557	0.0328	1.60[1.04-2.46]
Blood cancer	Overall survival	158	GSE4475	209803_s_at	0.0316	0.52[0.28-0.94]

Brain cancer	Overall survival	70	GSE7696	209803_s_at	0.0356	0.82[0.69-0.99]
Brain cancer	Overall survival	50	MGH-glioma	31888_s_at	0.0001	1.67[1.27-2.18]
Brain cancer	Overall survival	74	GSE4412-GPL96	209803_s_at	0.0128	1.46[1.08-1.97]
Brain cancer	Overall survival	67	GSE16581	229494_s_at	0.0299	2193.45[2.11-2276203.77]
Breast cancer	Overall survival	158	GSE3143	31888_s_at	0.0455	1.61[1.01-2.57]
Breast cancer	Overall survival	158	GSE3143	40237_at	0.0047	2.01[1.24-3.25]
Breast cancer	Distant metastasis-free survival	200	GSE11121	209802_at	0.0196	2.01[1.12-3.60]
Breast cancer	Distant metastasis-free survival	286	GSE2034	209803_s_at	0.0002	1.54[1.23-1.94]
Breast cancer	Disease Specific survival	159	GSE1456-GPL96	209803_s_at	0.0085	1.79[1.16-2.77]
Breast cancer	Overall survival	159	GSE1456-GPL96	209803_s_at	0.0034	1.74[1.20-2.53]
Breast cancer	Disease Specific survival	236	GSE3494-GPL96	209803_s_at	0.0281	1.50[1.04-2.14]
Breast cancer	Disease free survival	249	GSE4922-GPL96	209803_s_at	0.0201	1.42[1.06-1.91]
Breast cancer	Distant metastasis-free survival	125	GSE2990	209803_s_at	0.0074	1.55[1.12-2.12]
Breast cancer	Relapse-free survival	125	GSE2990	209803_s_at	0.0161	1.36[1.06-1.74]

Breast cancer	Distant metastasis-free survival	198	GSE7390	209803_s_at	0.0264	1.28[1.03-1.58]
Breast cancer	Overall survival	198	GSE7390	209803_s_at	0.0391	1.27[1.01-1.59]
Colorectal cancer	Overall survival	62	GSE12945	209803_s_at	0.0312	2.26[1.08-4.74]
Eye cancer	Distant metastasis-free survival	63	GSE22138	209803_s_at	0.0464	1.23[1.00-1.52]
Lung cancer	Overall survival	86	MICHIGAN-LC	AF001294_at	0.0081	3.79[1.39-10.28]
Lung cancer	Overall survival	204	GSE31210	209803_s_at	0.0021	2.36[1.37-4.06]
Lung cancer	Overall survival	204	GSE31210	229494_s_at	0.0444	1.45[1.01-2.08]
Lung cancer	Relapse-free survival	204	GSE31210	209803_s_at	0.0026	1.86[1.24-2.80]
Lung cancer	Relapse-free survival	204	GSE31210	229494_s_at	0.0404	1.31[1.01-1.70]
Lung cancer	Relapse-free survival	204	GSE31210	209802_at	0.0330	1.89[1.05-3.04]
Lung cancer	Disease Specific survival	90	GSE14814	209802 _at	0.0367	6.40[1.12-36.55]
Lung cancer	Relapse-free survival	138	GSE8894	209803_s_at	0.0279	1.24[1.02-1.51]
Ovarian cancer	Overall survival	278	GSE9891	229494_s_at	0.0205	0.15[0.03-0.75]
Ovarian cancer	Overall survival	133	DUKE-OC	209802_at	0.0095	4.19[1.42-12.38]
Ovarian cancer	Overall survival	80	GSE14764	209802_at	0.0394	1.77[1.03-3.06]

Supplementary Table 6. Association of PHLDA3 expression and survival of cancer patients (PrognoScan database).

Cancer type	Endpoint	N	Dataset	Probe ID	Cox P -value	HR [95% CI^{low}-CI^{upp}]
Breast cancer	Relapse-free survival	204	GSE7390	218634_at	0.0059	0.58[0.39-0.85]
Breast cancer	Distant metastasis - free survival	286	GSE2034	218634_at	0.0221	0.51[0.28-0.91]
Colorectal cancer	Disease free survival	226	GSE14333	218634_at	0.0291	1.85[1.06-3.22]
Lung cancer	Relapse-free survival	204	GSE31210	218634_at	0.0162	3.18[1.24-8.19
Skin cancer	Overall survival	38	GSE19234	218634_at	0.0296	2.29[1.09-4.84]

Supplementary Table S7. Alteration frequency of a ten-gene signature (PHLDA1, EIF3D, PLK2, DUSP6, RND3, MCL1, KLF6,SLC20A1, PABPC4, RPL14) in various cancers (cBioPortal web).

Cancer	Data source	N	Frequency (%)	Mutation % (N)	Amplification % (N)	Deletion % (N)	Multiple alterations % (N)
Bladder	TCGA, Cell2017	408	35.54%	8.09% (33)	20.59% (84)	2.94% (12)	3.92% (13)
Uterine	TCGA,	509	26.52%	14.93% (76)	10.02% (51)	0.98%(5cases)	0.59%(3cases)

	PanCancer Atlas						
Ovarian	TCGA, PanCancer Atlas	398	25.88%	2.26% (9)	20.1% (80)	2.51% (10)	1.01% (4)
Lung Adeno	TCGA, PanCancer Atlas	507	22.88%	6.11% (31)	12.82% (65)	2.56% (13)	1.38% (7)
Sarcoma	TCGA, PanCancer Atlas	253	21.74%	2.37% (16)	15.02% (38)	3.56% (9)	0.79% (2)
Skin	TCGA, PanCancer Atlas	363	21.49%	15.43% (56)	4.13% (15)	0.55% (2)	1.38% (5)
Esophageal Adeno	TCGA, PanCancer Atlas	182	19.23%	2.2% (4)	12.09% (22)	4.4% (8)	0.55% (1)
Stomach	TCGA, PanCancer Atlas	434	18.89%	8.29% (36)	7.14% (31)	3.23% (14)	0.23% (1)
Breast	TCGA, Nature 2012	996	18.27%	2.41% (24)	14.06% (140)	1.31% (13)	0.5% (5)
Liver	TCGA, Cell 2015	353	15.02%	0.62% (2)	2.4% (8)	11.71% (39)	0.3% (1)
Lymphoma	TCGA, PanCancer Atlas	37	13.51%	8.11% (3)	2.7% (1)	-	2.7% (1)
Prostate	TCGA, PanC	489	12.68%	1.23% (6)	3.48% (17)	7.77% (38)	0.2% (1)

	ancer Atlas						
Uterine	TCGA, PanCancer Atlas	56	12.5%	1.79% (1)	10.71 (16)	-	-
Adrenocortical	TCGA, PanCancer Atlas	89	12.36%	-	11.24% (10)	-	1.12% (1)
Cholorectal	TCGA, PanCancer Atlas	526	11.26%	8.94% (47)	1.33% (7)	0.95% (5)	0.38% (2)
Cholangiocarcinoma	TCGA, PanCancer Atlas	36	11.11%	-	8.33% (3)	2.78% (1)	-
Head and Neck Squ	TCGA, PanCancer Atlas	496	10.89%	4.84% (24)	3.02% (15)	2.42% (12)	0.6% (3)
Pheochromocytoma and Paraganglima	TCGA, PanCancer Atlas	161	9.94%	0.62% (1)	6.83% (11)	2.48% (4)	-
Pancreatic	TCGA, PanCancer Atlas	175	8.57%	2.87% (5)	5.14% (9)	0.57% (1)	-
Cervical Squ	TCGA, PanCancer Atlas	278	8.27%	4.68%(13)	2.88% (8)	-	0.72% (2)
Kidney	TCGA, PanCancer Atlas	274	6.93%	6.2% (17)	0.36% (1)	0.36% (1)	-
Thymoma	TCGA, PanC	123	5.69%	2.44% (3)	0.81% (1)	2.44% (3)	-

	ancer Atlas						
Glioblastoma	TCGA, PanCancer Atlas	378	5.56%	3.17% (12)	1.85% (1)	0.26% (1)	0.26% (1)
Brain	TCGA, PanCancer Atlas	511	5.28%	1.17% (6)	2.35% (12)	1.17% (9)	-
Testicular	TCGA, PanCancer Atlas	144	4.17%	-	1.39% (2)	2.78% (4)	-
Kidney Renal	TCGA, PanCancer Atlas	354	3.67%	0.855% (3)	0.56% (2)	2.26% (8)	-
Kidney Chro	TCGA, PanCancer Atlas	65	3.08%	-	3.08%(2)	-	-
Mesothelioma	TCGA, Cancer Cell 2014	82	2.44%	-	1.22% (1)	1.22% (1)	-
Leukemia	TCGA, PanCancer Atlas	190	1.58%	1.58% (3)	-	-	-
Thyroid	TCGA, PanCancer Atlas	482	1.04%	1.04% (5)			

Supplementary Table S8. Alteration frequency of a ten-gene signature (PHLDA2, PHLDA3, DUSP6, TAGLN2, BAMBI, SLC19A1, UPP1, FOSL1, S100P, MAFF) in various cancer (cBioPortal web).

Cancer	Data source	N	Frequency (%)	Mutation % (N)	Amplification % (N)	Deletion % (N)	Multiple alterations % (N)
Bladder	TCGA, PanCancer Atlas	406	26.11%	5.91% (24)	16.5% (67)	2.71% (11)	0.99% (4)
Uterine	TCGA, PanCancer Atlas	509	22%	11.79% (60)	9.43% (48)	0.79% (4)	-
Esophageal	TCGA, PanCancer Atlas	182	20.88%	4.4% (8)	10.99% (20)	5.49% (10)	-
Lung	TCGA, PanCancer Atlas	507	19.92%	4.73% (24)	12.03% (61)	1.97% (10)	1.18% (6)
Breast	TCGA, PanCancer Atlas	996	19.08%	1.2% (12)	16.06% (160)	1.61% (16)	0.2% (2)
Ovarian	TCGA, PanCancer Atlas	398	18.34%	0.5% (2)	15.08% (60)	2.01% (8)	0.75% (3)
Melanoma Skin	TCGA, PanCancer Atlas	363	17.91%	6.06% (22)	10.19% (37)	1.38% (5)	0.28% (1)
Sarcoma	TCGA, PanCancer Atlas	253	17.79%	0.79% (2)	13.44% (34)	3.16% (8)	0.4% (1)
Liver	TCGA, PanCancer Atlas	353	17%	1.98% (7)	13.88% (49)	1.13% (14)	-

Lung Squ	TCGA, PanCancer Atlas	469	14.5%	2.99% (14)	8.53% (40)	2.13% (10)	0.85% (4)
Head and Neck Squ	TCGA, PanCancer Atlas	496	13.91%	3.43% (17)	7.66% (38)	2.22% (11)	0.6% (3)
Cholangiocarcinoma	TCGA, PanCancer Atlas	36	13.89%	-	13.89% (5)	-	-
Stomach	TCGA, PanCancer Atlas	434	12.9%	4.38% (19)	4.38% (19)	3.69% (16)	0.42% (2)
Uterine	TCGA, PanCancer Atlas	56	12.5%	1.79% (1)	10.71% (6)	-	-
Adrenocortical	TCGA, PanCancer Atlas	89	12.36%	1.12% (1)	7.87% (	3.37% (3)	-
Pancreatic	TCGA, PanCancer Atlas	175	9.14%	2.29% (4)	6.29% (11)	0.57% (1)	-
Cervical	TCGA, PanCancer Atlas	278	8.99%	2.52% (7)	4.68% (13)	1.44% (4)	0.36% (1)
Brain	TCGA, PanCancer Atlas	511	8.02%	0.59% (3)	3.52% (18)	3.91% (20)	-
Colorectal	TCGA, PanCancer Atlas	526	7.98%	6.08% (32)	0.95% (5)	0.95% (5)	-
Pheochromocytoma and Paraganglima	TCGA, PanCancer Atlas	161	7.45%	-	6.83% (11)	0.62% (1)	-

Prostate	TCGA, PanCancer Atlas	489	7.36%	1.02% (5)	3.48% (17)	2.86% (14)	-
Glioblastoma Multi	TCGA, PanCancer Atlas	378	6.88%	1.85% (7)	3.7% (14)	1.06% (4)	0.26% (1)
Lymphoma	TCGA, PanCancer Atlas	37	5.41%	2.7% (1)	2.7% (1)	-	-
Testicular	TCGA, PanCancer Atlas	144	4.17%	1.39% (2)	0.69% (1)	2.08% (3)	-
Thymoma	TCGA, PanCancer Atlas	123	4.07%	-	1.63% (2)	2.44% (3)	-
Kidney	TCGA, PanCancer Atlas	274	4.01%	2.19% (6)	1.09% (3)	0.73% (2)	-
Melanoma	TCGA, PanCancer Atlas	80	2.5%	1.25% (1)	1.25% (1)	-	-
Mesothelioma	TCGA, PanCancer Atlas	82	2.44%	1.22% (1)	1.22% (1)	-	-
Leukemia	TCGA, PanCancer Atlas	198	2.11%	-	2.11%(4)	-	-
Kidney	TCGA, Nature 2013	65	1.54%	1.54% (1)	-	-	-
Kidney	TCGA, cell 2014	354	1.13%	0.28% (1)	0.28% (1)	0.56% (2)	-
Thyroid	TCGA, cell 2014	482	0.83%	0.41% (2)	0.41% (2)	-	-

.

Supplementary Table S9. Alteration frequency of a ten-gene signature (PHLDA3, PHLDA2, DYNLL1, NPAS1, RARS2, DNAJB2, GSN, ZMAT3, ME1, SAAL1) in various cancer (cBioPortal web).

**Cancer**	**Data source**	**N**	**Frequency (%)**	**Mutation % (N)**	**Amplification % (N)**	**Deletion % (N)**	**Multiple alterations % (N)**
Lung Squ	TCGA, PanCancer Atlas	469	43.07%	3.2% (15)	36.25% (170)	1.49% (7)	2.13% (10)
Uterine	TCGA, PanCancer Atlas	509	29.27%	16.31% (83)	10.61% (54)	1.18% (6)	1.18% (6)
Ovarian	TCGA, PanCancer Atlas	398	27.64%	1.01% (4)	24.87% (99)	1.26% (5)	0.5% (2)
Esophageal	TCGA, PanCancer Atlas	182	26.37%	2.75% (5)	19.23% (35)	3.85% (7)	0.55% (1)
Skin	TCGA, PanCancer Atlas	363	26.17%	18.73% (68)	4.41% (16)	1.65% (6)	1.38% (5)
Head and Neck Squ	TCGA, PanCancer Atlas	496	22.18%	4.23% (21)	15.52% (77)	1.61% (8)	0.81% (4)
Cervical	TCGA, PanCancer Atlas	278	20.5%	3.24% (9)	14.39% (40)	1.8% (5)	1.08% (3)
Uterine	TCGA, PanCancer Atlas	56	19.64%	5.36% (3)	14.29% (8)	-	-
Bladder	TCGA, PanCancer Atlas	406	17.98%	7.14% (29)	6.4% (26)	3.94% (16)	0.49% (2)

Prostate	TCGA, PanCancer Atlas	489	17.38%	1.23% (6)	4.7% (23)	11.45% (56)	-
Lymphoma	TCGA, PanCancer Atlas	37	16.22%	5.41% (2)	2.7% (1)	8.11% (3)	-
Breast	TCGA, PanCancer Atlas	996	16.16%	1.61% (16)	12.55% (125)	1.81% (18)	0.2% (2)
Stomach	TCGA, PanCancer Atlas	434	16.13%	6.68% (29)	6.91% (30)	2.3% (10)	0.23% (1)
Lung	TCGA, PanCancer Atlas	507	15.98%	7.3% (37)	5.92% (30)	2.17% (11)	0.59% (3)
Liver	TCGA, PanCancer Atlas	353	12.46%	1.42% (4)	8.22% (29)	2.27% (8)	0.57% (2)
Sarcoma	TCGA, PanCancer Atlas	253	11.86%	1.98% (5)	7.11% (18)	2.77% (7)	-
Carcinoma	TCGA, PanCancer Atlas	89	11.24%	4.49% (4)	6.74% (6)	-	-
Colorectal	TCGA, PanCancer Atlas	526	10.46%	9.32% (49)	0.76% (4)	0.38% (2)	-
Melanoma	TCGA, PanCancer Atlas	80	8.75%	1.25% (1)	2.5% (2)	5% (4)	-
Brain	TCGA, PanCancer Atlas	511	8.61%	0.78% (4)	2.54% (13)	5.09% (26)	0.2% (1)
Cholangiocarcinoma	TCGA, PanCancer Atlas	36	8.33%	2.78% (1)	5.56% (2)	-	-

Glioblastoma	TCGA, PanCancer Atlas	378	7.41%	2.65% (10)	4.5% (17)	0.26% (1)	-
Kidney	TCGA, PanCancer Atlas	274	6.57%	3.65% (10)	1.82% (5)	1.09% (3)	-
Pheochromocytoma and paraganglioma	TCGA, PanCancer Atlas	161	5.59%	4.35% (7)	1.24% (2)	-	-
Kidney	TCGA, PanCancer Atlas	354	4.24%	1.41% (5)	1.69% (6)	0.85% (3)	0.28% (1)
Testicular	TCGA, PanCancer Atlas	144	4.17%	2.08% (3)	1.39% (2)	0.69% (1)	-
Thymoma	TCGA, PanCancer Atlas	123	4.07%	2.44% (3)	1.63% (2)	-	-
Mesothelioma	TCGA, PanCancer Atlas	82	3.66%	1.22% (1)	2.44% (2)	-	-
Kidney	TCGA, PanCancer Atlas	65	3.08%	1.54% (1)	1.54% (1)	-	-
Leukemia	TCGA, PanCancer Atlas	190	2.11% (4)	-	-	-	--
Thyroid	TCGA, PanCancer Atlas	482	1.87%	0.83% (4)	0.21% (1)	0.83% (4)	

Supplementary Figures

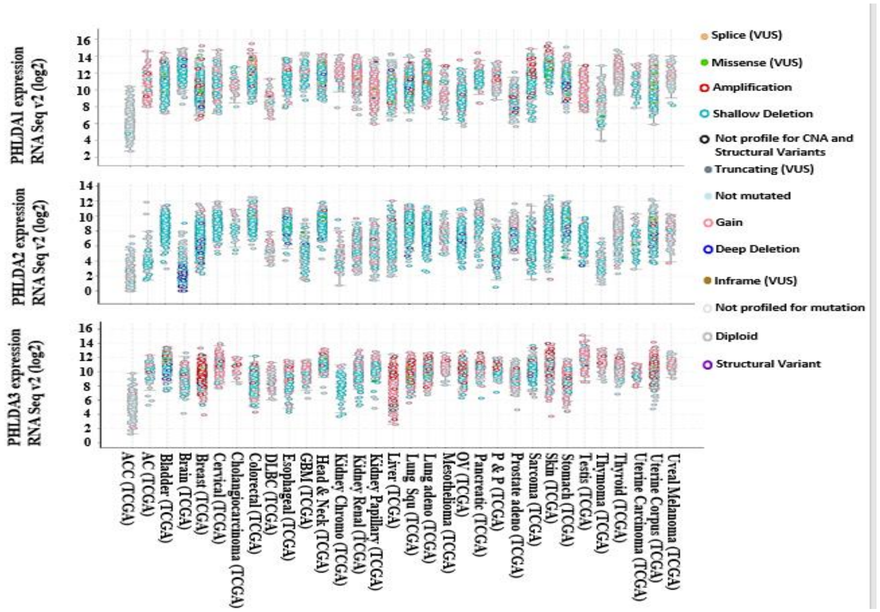

Supplementary Figure S1. The cBioPortal database (http://www.cbioportal.org/index.do) was analyzed to determine the expression of PHLDA family genes in 32 different forms of human cancer. Each dot corresponds to a distinct study. Green dots denote missense mutations, blue dots represent typical gene sequencing results (no mutation), and white dots indicate those without profiled data. Each box displays the median and interquartile ranges.

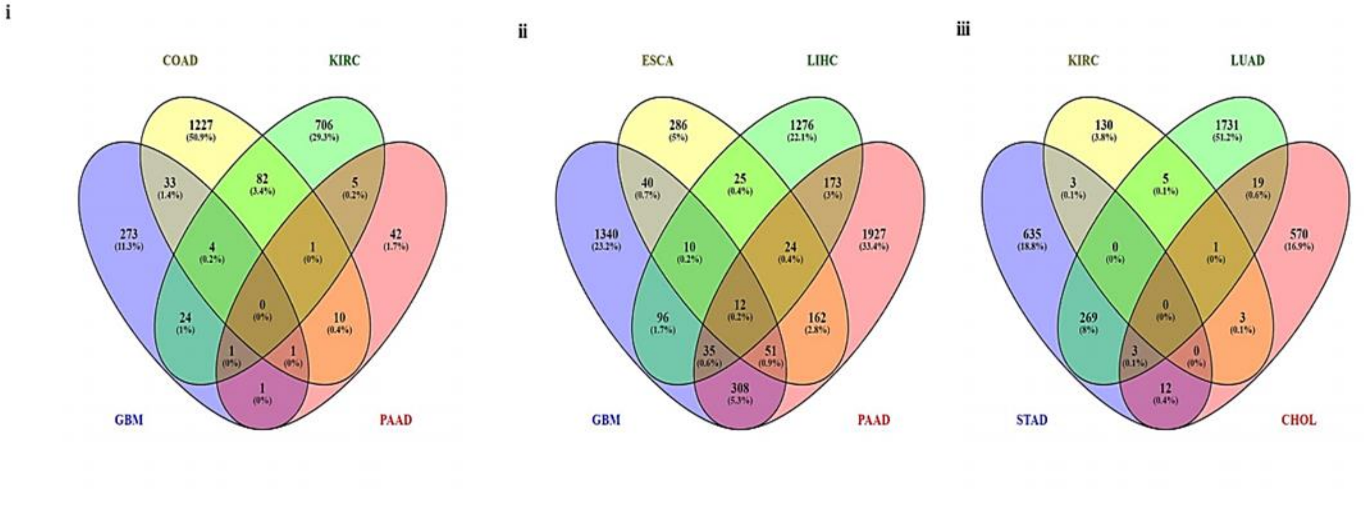

Supplementary Figure S2. Venn diagram shows the coincident genes with PHLDA1(i), PHLDA2 (ii), and PHLDA3 (iii) that are negatively associated.

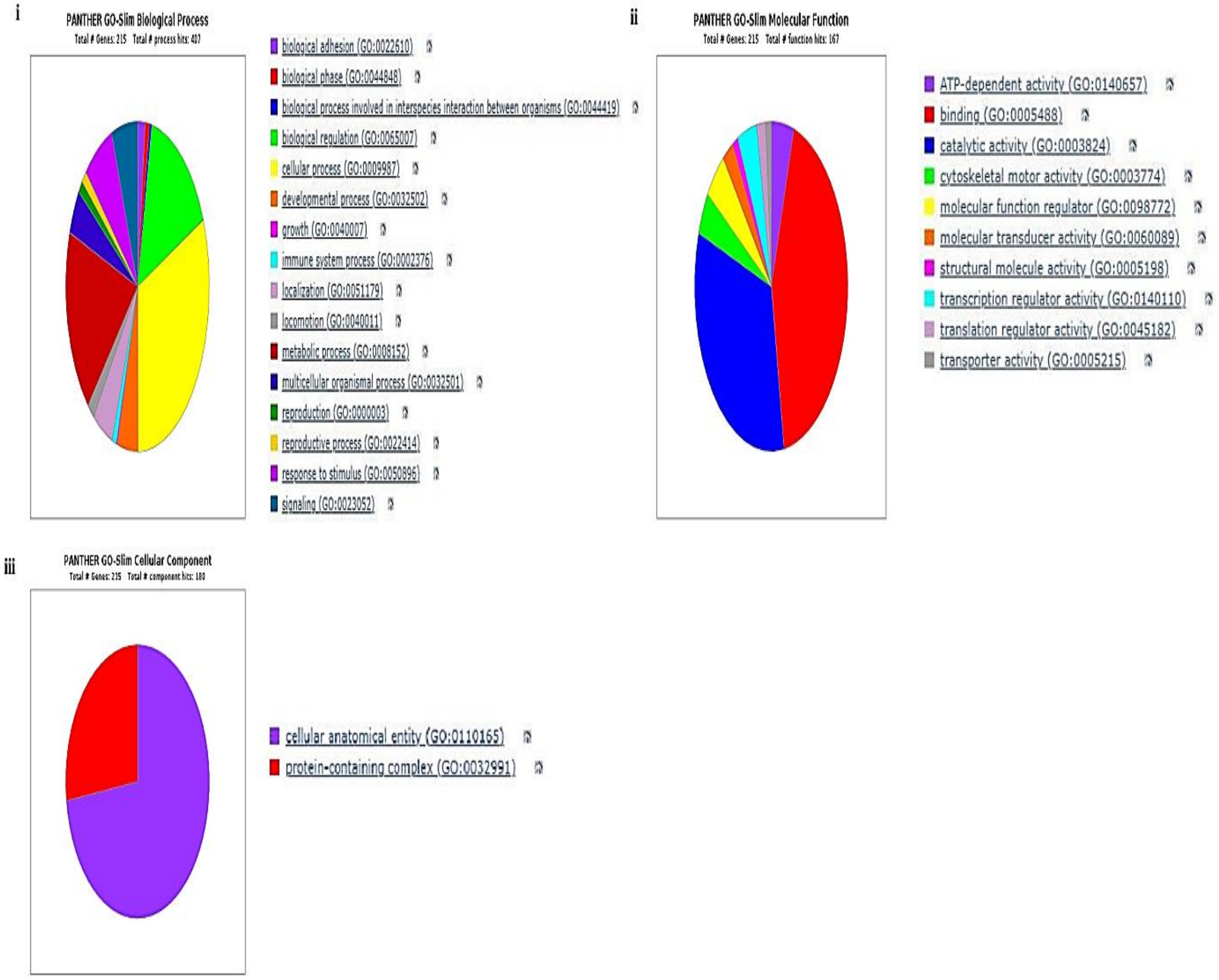

Supplementary Figure S3. Gene ontology (GO) analysis of positively correlated genes of *PHLDA1* using PANTHER. Here, Molecular function (**i**), Biological Process (**ii**), and Cellular component (**iii**).

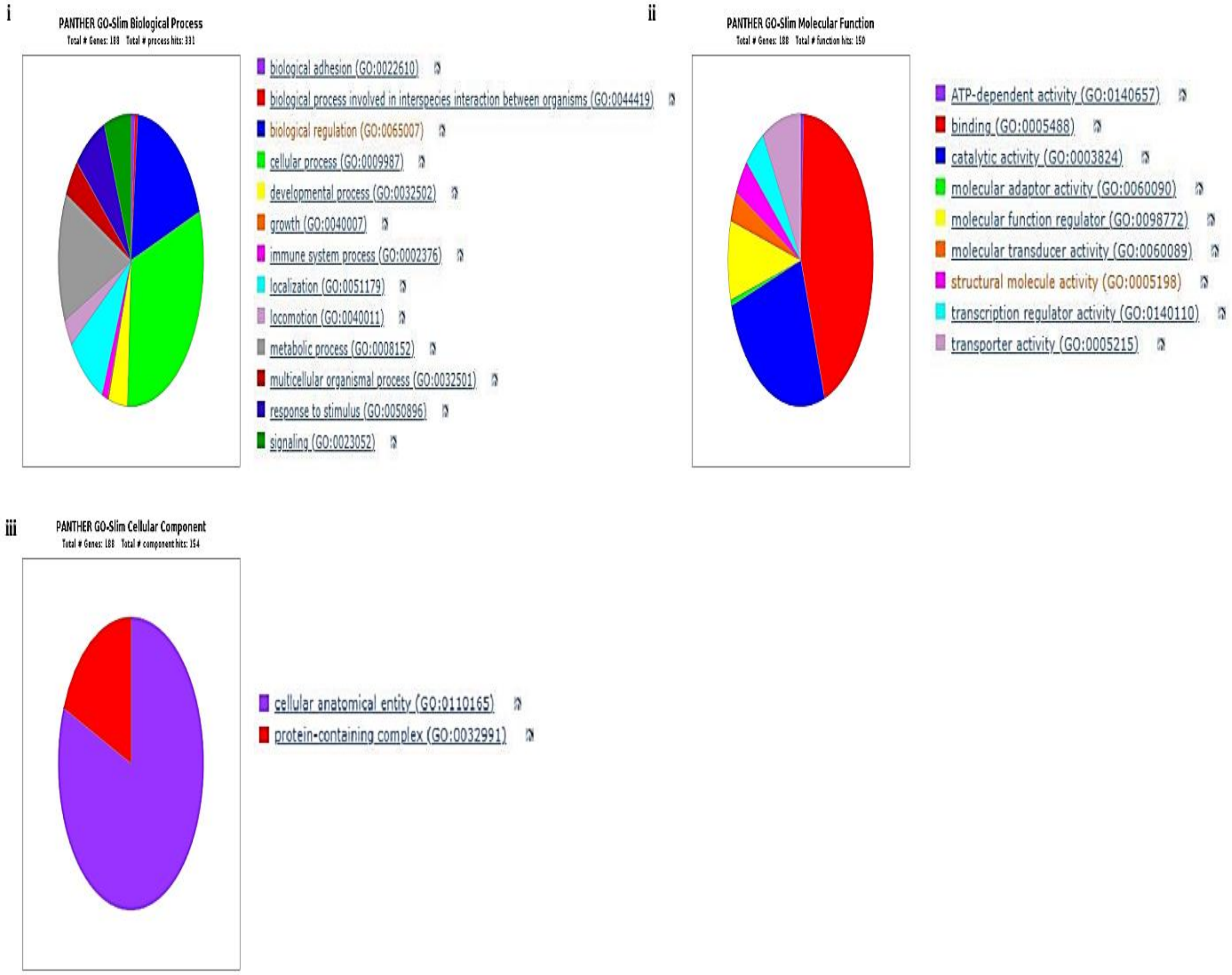

Supplementary Figure S4. Gene ontology (GO) analysis of positively correlated genes of *PHLDA2* using PANTHER. Here, Molecular function (**i**), Biological Process (**ii**), and Cellular component (**iii**).

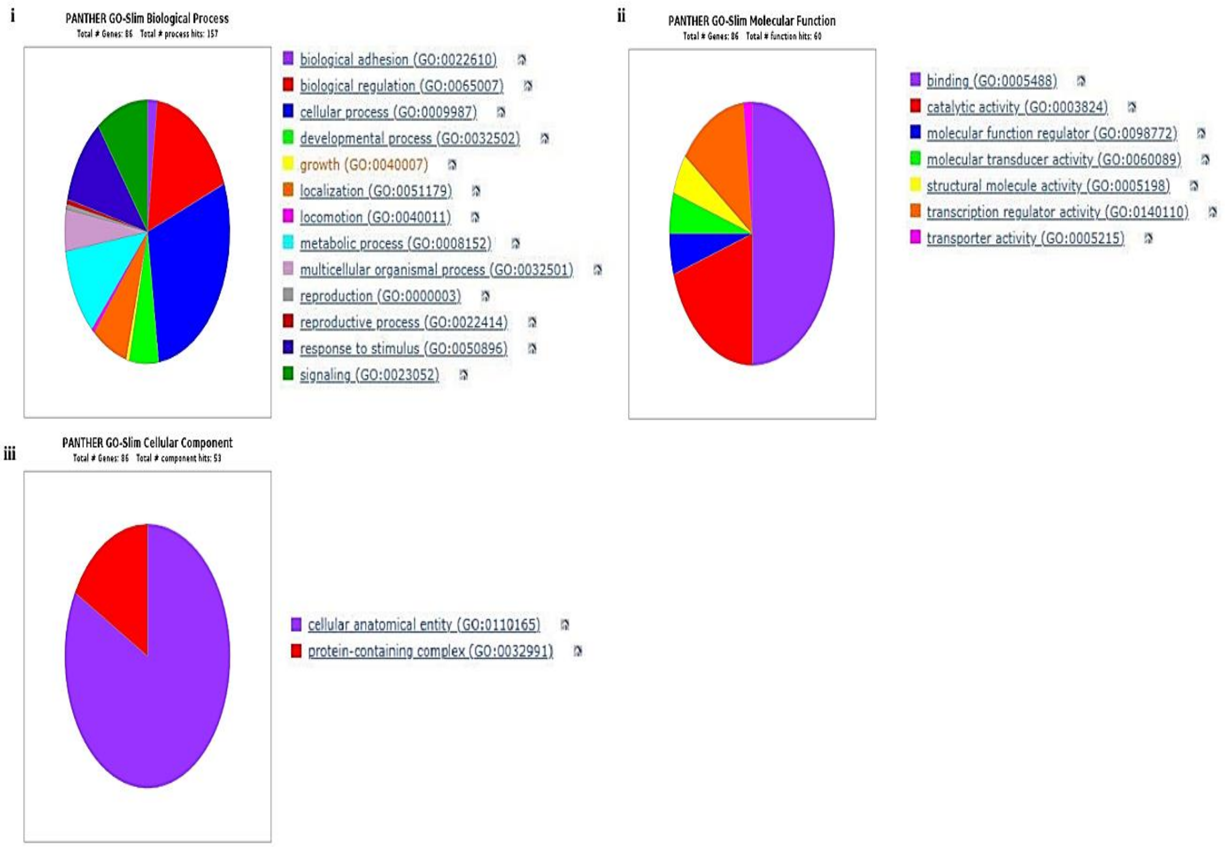

Supplementary Figure S5. Gene ontology (GO) analysis of positively correlated genes of *PHLDA3* using PANTHER. Here, Molecular function (**i**), Biological Process (**ii**), and Cellular component (**iii**).

References:

Agnelli, L., Mosca, L., Fabris, S., Lionetti, M., Andronache, A., Kwee, I., Todoerti, K., Verdelli, D., Battaglia, C., Bertoni, F., Deliliers, G. L., & Neri, A. (2009). A SNP microarray and FISH-based procedure to detect allelic imbalances in multiple myeloma: an integrated genomics approach reveals a wide gene dosage effect. *Genes Chromosomes Cancer*, *48*(7), 603-614. https://doi.org/10.1002/gcc.20668

Badea, L., Herlea, V., Dima, S. O., Dumitrascu, T., & Popescu, I. (2008). Combined gene expression analysis of whole-tissue and microdissected pancreatic ductal adenocarcinoma identifies genes specifically overexpressed in tumor epithelia. *Hepatogastroenterology*, *55*(88), 2016-2027.

Barretina, J., Taylor, B. S., Banerji, S., Ramos, A. H., Lagos-Quintana, M., Decarolis, P. L., Shah, K., Socci, N. D., Weir, B. A., Ho, A., Chiang, D. Y., Reva, B., Mermel, C. H., Getz, G., Antipin, Y., Beroukhim, R., Major, J. E., Hatton, C., Nicoletti, R., . . . Singer, S. (2010). Subtype-specific genomic alterations define new targets for soft-tissue sarcoma therapy. *Nat Genet*, *42*(8), 715-721. https://doi.org/10.1038/ng.619

Basso, K., Margolin, A. A., Stolovitzky, G., Klein, U., Dalla-Favera, R., & Califano, A. (2005). Reverse engineering of regulatory networks in human B cells. *Nat Genet*, *37*(4), 382-390. https://doi.org/10.1038/ng1532

Beer, D. G., Kardia, S. L., Huang, C. C., Giordano, T. J., Levin, A. M., Misek, D. E., Lin, L., Chen, G., Gharib, T. G., Thomas, D. G., Lizyness, M. L., Kuick, R., Hayasaka, S., Taylor, J. M., Iannettoni, M. D., Orringer, M. B., & Hanash, S. (2002). Gene-expression profiles predict survival of patients with lung adenocarcinoma. *Nat Med*, *8*(8), 816-824. https://doi.org/10.1038/nm733

Bonome, T., Levine, D. A., Shih, J., Randonovich, M., Pise-Masison, C. A., Bogomolniy, F., Ozbun, L., Brady, J., Barrett, J. C., Boyd, J., & Birrer, M. J. (2008). A gene signature predicting for survival in suboptimally debulked patients with ovarian cancer. *Cancer Res*, *68*(13), 5478-5486. https://doi.org/10.1158/0008-5472.Can-07-6595

Bredel, M., Bredel, C., Juric, D., Harsh, G. R., Vogel, H., Recht, L. D., & Sikic, B. I. (2005). Functional network analysis reveals extended gliomagenesis pathway maps and three novel MYC-interacting genes in human gliomas. *Cancer Res*, *65*(19), 8679-8689. https://doi.org/10.1158/0008-5472.Can-05-1204

Brune, V., Tiacci, E., Pfeil, I., Döring, C., Eckerle, S., van Noesel, C. J., Klapper, W., Falini, B., von Heydebreck, A., Metzler, D., Bräuninger, A., Hansmann, M. L., & Küppers, R. (2008). Origin and pathogenesis of nodular lymphocyte-predominant Hodgkin lymphoma as revealed by global gene expression analysis. *J Exp Med*, *205*(10), 2251-2268. https://doi.org/10.1084/jem.20080809

Chen, X., Cheung, S. T., So, S., Fan, S. T., Barry, C., Higgins, J., Lai, K. M., Ji, J., Dudoit, S., Ng, I. O., Van De Rijn, M., Botstein, D., & Brown, P. O. (2002). Gene expression patterns in human liver cancers. *Mol Biol Cell*, *13*(6), 1929-1939. https://doi.org/10.1091/mbc.02-02-0023

Cho, J. Y., Lim, J. Y., Cheong, J. H., Park, Y. Y., Yoon, S. L., Kim, S. M., Kim, S. B., Kim, H., Hong, S. W., Park, Y. N., Noh, S. H., Park, E. S., Chu, I. S., Hong, W. K., Ajani, J. A., & Lee, J. S. (2011). Gene expression signature-based prognostic risk score in gastric cancer. *Clin Cancer Res*, *17*(7), 1850-1857. https://doi.org/10.1158/1078-0432.Ccr-10-2180

Compagno, M., Lim, W. K., Grunn, A., Nandula, S. V., Brahmachary, M., Shen, Q., Bertoni, F., Ponzoni, M., Scandurra, M., Califano, A., Bhagat, G., Chadburn, A., Dalla-Favera, R., & Pasqualucci, L. (2009). Mutations of multiple genes cause deregulation of NF-kappaB in diffuse large B-cell lymphoma. *Nature*, *459*(7247), 717-721. https://doi.org/10.1038/nature07968

Estilo, C. L., P, O. c., Talbot, S., Socci, N. D., Carlson, D. L., Ghossein, R., Williams, T., Yonekawa, Y., Ramanathan, Y., Boyle, J. O., Kraus, D. H., Patel, S., Shaha, A. R., Wong, R. J., Huryn, J. M., Shah, J. P., & Singh, B. (2009). Oral tongue cancer gene expression profiling: Identification of novel potential prognosticators by oligonucleotide microarray analysis. *BMC Cancer*, *9*, 11. https://doi.org/10.1186/1471-2407-9-11

French, P. J., Swagemakers, S. M., Nagel, J. H., Kouwenhoven, M. C., Brouwer, E., van der Spek, P., Luider, T. M., Kros, J. M., van den Bent, M. J., & Sillevis Smitt, P. A. (2005). Gene expression profiles associated with treatment response in oligodendrogliomas. *Cancer Res*, *65*(24), 11335-11344. https://doi.org/10.1158/0008-5472.Can-05-1886

Gaedcke, J., Grade, M., Jung, K., Camps, J., Jo, P., Emons, G., Gehoff, A., Sax, U., Schirmer, M., Becker, H., Beissbarth, T., Ried, T., & Ghadimi, B. M. (2010). Mutated KRAS results in overexpression of DUSP4, a MAP-kinase phosphatase, and SMYD3, a histone methyltransferase, in rectal carcinomas. *Genes Chromosomes Cancer*, *49*(11), 1024-1034. https://doi.org/10.1002/gcc.20811

Gaspar, C., Cardoso, J., Franken, P., Molenaar, L., Morreau, H., Möslein, G., Sampson, J., Boer, J. M., de Menezes, R. X., & Fodde, R. (2008). Cross-species comparison of human and mouse intestinal polyps reveals conserved mechanisms in adenomatous polyposis coli (APC)-driven tumorigenesis. *Am J Pathol*, *172*(5), 1363-1380. https://doi.org/10.2353/ajpath.2008.070851

Haqq, C., Nosrati, M., Sudilovsky, D., Crothers, J., Khodabakhsh, D., Pulliam, B. L., Federman, S., Miller, J. R., 3rd, Allen, R. E., Singer, M. I., Leong, S. P., Ljung, B. M., Sagebiel, R. W., &

Kashani-Sabet, M. (2005). The gene expression signatures of melanoma progression. *Proc Natl Acad Sci U S A*, *102*(17), 6092-6097. https://doi.org/10.1073/pnas.0501564102
Hendrix, N. D., Wu, R., Kuick, R., Schwartz, D. R., Fearon, E. R., & Cho, K. R. (2006). Fibroblast growth factor 9 has oncogenic activity and is a downstream target of Wnt signaling in ovarian endometrioid adenocarcinomas. *Cancer Res*, *66*(3), 1354-1362. https://doi.org/10.1158/0008-5472.Can-05-3694
Higgins, J. P., Shinghal, R., Gill, H., Reese, J. H., Terris, M., Cohen, R. J., Fero, M., Pollack, J. R., van de Rijn, M., & Brooks, J. D. (2003). Gene expression patterns in renal cell carcinoma assessed by complementary DNA microarray. *Am J Pathol*, *162*(3), 925-932. https://doi.org/10.1016/s0002-9440(10)63887-4
Hong, Y., Downey, T., Eu, K. W., Koh, P. K., & Cheah, P. Y. (2010). A 'metastasis-prone' signature for early-stage mismatch-repair proficient sporadic colorectal cancer patients and its implications for possible therapeutics. *Clin Exp Metastasis*, *27*(2), 83-90. https://doi.org/10.1007/s10585-010-9305-4
Hu, N., Clifford, R. J., Yang, H. H., Wang, C., Goldstein, A. M., Ding, T., Taylor, P. R., & Lee, M. P. (2010). Genome wide analysis of DNA copy number neutral loss of heterozygosity (CNNLOH) and its relation to gene expression in esophageal squamous cell carcinoma. *BMC Genomics*, *11*, 576. https://doi.org/10.1186/1471-2164-11-576
Jones, J., Otu, H., Spentzos, D., Kolia, S., Inan, M., Beecken, W. D., Fellbaum, C., Gu, X., Joseph, M., Pantuck, A. J., Jonas, D., & Libermann, T. A. (2005). Gene signatures of progression and metastasis in renal cell cancer. *Clin Cancer Res*, *11*(16), 5730-5739. https://doi.org/10.1158/1078-0432.Ccr-04-2225
Kaiser, S., Park, Y. K., Franklin, J. L., Halberg, R. B., Yu, M., Jessen, W. J., Freudenberg, J., Chen, X., Haigis, K., Jegga, A. G., Kong, S., Sakthivel, B., Xu, H., Reichling, T., Azhar, M., Boivin, G. P., Roberts, R. B., Bissahoyo, A. C., Gonzales, F., . . . Aronow, B. J. (2007). Transcriptional recapitulation and subversion of embryonic colon development by mouse colon tumor models and human colon cancer. *Genome Biol*, *8*(7), R131. https://doi.org/10.1186/gb-2007-8-7-r131
Ki, D. H., Jeung, H. C., Park, C. H., Kang, S. H., Lee, G. Y., Lee, W. S., Kim, N. K., Chung, H. C., & Rha, S. Y. (2007). Whole genome analysis for liver metastasis gene signatures in colorectal cancer. *Int J Cancer*, *121*(9), 2005-2012. https://doi.org/10.1002/ijc.22975
Kim, S. M., Park, Y. Y., Park, E. S., Cho, J. Y., Izzo, J. G., Zhang, D., Kim, S. B., Lee, J. H., Bhutani, M. S., Swisher, S. G., Wu, X., Coombes, K. R., Maru, D., Wang, K. K., Buttar, N. S., Ajani, J. A., & Lee, J. S. (2010). Prognostic biomarkers for esophageal adenocarcinoma identified by analysis of tumor transcriptome. *PLoS One*, *5*(11), e15074. https://doi.org/10.1371/journal.pone.0015074
Korkola, J. E., Houldsworth, J., Chadalavada, R. S., Olshen, A. B., Dobrzynski, D., Reuter, V. E., Bosl, G. J., & Chaganti, R. S. (2006). Down-regulation of stem cell genes, including those in a 200-kb gene cluster at 12p13.31, is associated with in vivo differentiation of human male germ cell tumors. *Cancer Res*, *66*(2), 820-827. https://doi.org/10.1158/0008-5472.Can-05-2445
Landi, M. T., Dracheva, T., Rotunno, M., Figueroa, J. D., Liu, H., Dasgupta, A., Mann, F. E., Fukuoka, J., Hames, M., Bergen, A. W., Murphy, S. E., Yang, P., Pesatori, A. C., Consonni, D., Bertazzi, P. A., Wacholder, S., Shih, J. H., Caporaso, N. E., & Jen, J. (2008). Gene expression signature of cigarette smoking and its role in lung adenocarcinoma development and survival. *PLoS One*, *3*(2), e1651. https://doi.org/10.1371/journal.pone.0001651
Lee, J., Kotliarova, S., Kotliarov, Y., Li, A., Su, Q., Donin, N. M., Pastorino, S., Purow, B. W., Christopher, N., Zhang, W., Park, J. K., & Fine, H. A. (2006). Tumor stem cells derived from glioblastomas cultured in bFGF and EGF more closely mirror the phenotype and genotype of primary tumors than do serum-cultured cell lines. *Cancer Cell*, *9*(5), 391-403. https://doi.org/10.1016/j.ccr.2006.03.030
Lu, K. H., Patterson, A. P., Wang, L., Marquez, R. T., Atkinson, E. N., Baggerly, K. A., Ramoth, L. R., Rosen, D. G., Liu, J., Hellstrom, I., Smith, D., Hartmann, L., Fishman, D., Berchuck, A., Schmandt, R., Whitaker, R., Gershenson, D. M., Mills, G. B., & Bast, R. C., Jr. (2004). Selection

of potential markers for epithelial ovarian cancer with gene expression arrays and recursive descent partition analysis. *Clin Cancer Res*, *10*(10), 3291-3300. https://doi.org/10.1158/1078-0432.Ccr-03-0409

Ma, X. J., Dahiya, S., Richardson, E., Erlander, M., & Sgroi, D. C. (2009). Gene expression profiling of the tumor microenvironment during breast cancer progression. *Breast Cancer Res*, *11*(1), R7. https://doi.org/10.1186/bcr2222

Murat, A., Migliavacca, E., Gorlia, T., Lambiv, W. L., Shay, T., Hamou, M. F., de Tribolet, N., Regli, L., Wick, W., Kouwenhoven, M. C., Hainfellner, J. A., Heppner, F. L., Dietrich, P. Y., Zimmer, Y., Cairncross, J. G., Janzer, R. C., Domany, E., Delorenzi, M., Stupp, R., & Hegi, M. E. (2008). Stem cell-related "self-renewal" signature and high epidermal growth factor receptor expression associated with resistance to concomitant chemoradiotherapy in glioblastoma. *J Clin Oncol*, *26*(18), 3015-3024. https://doi.org/10.1200/jco.2007.15.7164

Pei, H., Li, L., Fridley, B. L., Jenkins, G. D., Kalari, K. R., Lingle, W., Petersen, G., Lou, Z., & Wang, L. (2009). FKBP51 affects cancer cell response to chemotherapy by negatively regulating Akt. *Cancer Cell*, *16*(3), 259-266. https://doi.org/10.1016/j.ccr.2009.07.016

Piccaluga, P. P., Agostinelli, C., Califano, A., Rossi, M., Basso, K., Zupo, S., Went, P., Klein, U., Zinzani, P. L., Baccarani, M., Dalla Favera, R., & Pileri, S. A. (2007). Gene expression analysis of peripheral T cell lymphoma, unspecified, reveals distinct profiles and new potential therapeutic targets. *J Clin Invest*, *117*(3), 823-834. https://doi.org/10.1172/jci26833

Sabates-Bellver, J., Van der Flier, L. G., de Palo, M., Cattaneo, E., Maake, C., Rehrauer, H., Laczko, E., Kurowski, M. A., Bujnicki, J. M., Menigatti, M., Luz, J., Ranalli, T. V., Gomes, V., Pastorelli, A., Faggiani, R., Anti, M., Jiricny, J., Clevers, H., & Marra, G. (2007). Transcriptome profile of human colorectal adenomas. *Mol Cancer Res*, *5*(12), 1263-1275. https://doi.org/10.1158/1541-7786.Mcr-07-0267

Scotto, L., Narayan, G., Nandula, S. V., Arias-Pulido, H., Subramaniyam, S., Schneider, A., Kaufmann, A. M., Wright, J. D., Pothuri, B., Mansukhani, M., & Murty, V. V. (2008). Identification of copy number gain and overexpressed genes on chromosome arm 20q by an integrative genomic approach in cervical cancer: potential role in progression. *Genes Chromosomes Cancer*, *47*(9), 755-765. https://doi.org/10.1002/gcc.20577

Selamat, S. A., Chung, B. S., Girard, L., Zhang, W., Zhang, Y., Campan, M., Siegmund, K. D., Koss, M. N., Hagen, J. A., Lam, W. L., Lam, S., Gazdar, A. F., & Laird-Offringa, I. A. (2012). Genome-scale analysis of DNA methylation in lung adenocarcinoma and integration with mRNA expression. *Genome Res*, *22*(7), 1197-1211. https://doi.org/10.1101/gr.132662.111

Skrzypczak, M., Goryca, K., Rubel, T., Paziewska, A., Mikula, M., Jarosz, D., Pachlewski, J., Oledzki, J., & Ostrowski, J. (2010). Modeling oncogenic signaling in colon tumors by multidirectional analyses of microarray data directed for maximization of analytical reliability. *PLoS One*, *5*(10). https://doi.org/10.1371/journal.pone.0013091

Sperger, J. M., Chen, X., Draper, J. S., Antosiewicz, J. E., Chon, C. H., Jones, S. B., Brooks, J. D., Andrews, P. W., Brown, P. O., & Thomson, J. A. (2003). Gene expression patterns in human embryonic stem cells and human pluripotent germ cell tumors. *Proc Natl Acad Sci U S A*, *100*(23), 13350-13355. https://doi.org/10.1073/pnas.2235735100

Stearman, R. S., Dwyer-Nield, L., Zerbe, L., Blaine, S. A., Chan, Z., Bunn, P. A., Jr., Johnson, G. L., Hirsch, F. R., Merrick, D. T., Franklin, W. A., Baron, A. E., Keith, R. L., Nemenoff, R. A., Malkinson, A. M., & Geraci, M. W. (2005). Analysis of orthologous gene expression between human pulmonary adenocarcinoma and a carcinogen-induced murine model. *Am J Pathol*, *167*(6), 1763-1775. https://doi.org/10.1016/s0002-9440(10)61257-6

Su, H., Hu, N., Yang, H. H., Wang, C., Takikita, M., Wang, Q. H., Giffen, C., Clifford, R., Hewitt, S. M., Shou, J. Z., Goldstein, A. M., Lee, M. P., & Taylor, P. R. (2011). Global gene expression profiling and validation in esophageal squamous cell carcinoma and its association with clinical phenotypes. *Clin Cancer Res*, *17*(9), 2955-2966. https://doi.org/10.1158/1078-0432.Ccr-10-2724

Su, L. J., Chang, C. W., Wu, Y. C., Chen, K. C., Lin, C. J., Liang, S. C., Lin, C. H., Whang-Peng, J., Hsu, S. L., Chen, C. H., & Huang, C. Y. (2007). Selection of DDX5 as a novel internal control for Q-RT-PCR from microarray data using a block bootstrap re-sampling scheme. *BMC Genomics*, *8*, 140. https://doi.org/10.1186/1471-2164-8-140

Sun, L., Hui, A. M., Su, Q., Vortmeyer, A., Kotliarov, Y., Pastorino, S., Passaniti, A., Menon, J., Walling, J., Bailey, R., Rosenblum, M., Mikkelsen, T., & Fine, H. A. (2006). Neuronal and glioma-derived stem cell factor induces angiogenesis within the brain. *Cancer Cell*, *9*(4), 287-300. https://doi.org/10.1016/j.ccr.2006.03.003

Talantov, D., Mazumder, A., Yu, J. X., Briggs, T., Jiang, Y., Backus, J., Atkins, D., & Wang, Y. (2005). Novel genes associated with malignant melanoma but not benign melanocytic lesions. *Clin Cancer Res*, *11*(20), 7234-7242. https://doi.org/10.1158/1078-0432.Ccr-05-0683

Talbot, S. G., Estilo, C., Maghami, E., Sarkaria, I. S., Pham, D. K., P, O. c., Socci, N. D., Ngai, I., Carlson, D., Ghossein, R., Viale, A., Park, B. J., Rusch, V. W., & Singh, B. (2005). Gene expression profiling allows distinction between primary and metastatic squamous cell carcinomas in the lung. *Cancer Res*, *65*(8), 3063-3071. https://doi.org/10.1158/0008-5472.Can-04-1985

Wang, S., Zhan, M., Yin, J., Abraham, J. M., Mori, Y., Sato, F., Xu, Y., Olaru, A., Berki, A. T., Li, H., Schulmann, K., Kan, T., Hamilton, J. P., Paun, B., Yu, M. M., Jin, Z., Cheng, Y., Ito, T., Mantzur, C., . . . Meltzer, S. J. (2006). Transcriptional profiling suggests that Barrett's metaplasia is an early intermediate stage in esophageal adenocarcinogenesis. *Oncogene*, *25*(23), 3346-3356. https://doi.org/10.1038/sj.onc.1209357

Wurmbach, E., Chen, Y. B., Khitrov, G., Zhang, W., Roayaie, S., Schwartz, M., Fiel, I., Thung, S., Mazzaferro, V., Bruix, J., Bottinger, E., Friedman, S., Waxman, S., & Llovet, J. M. (2007). Genome-wide molecular profiles of HCV-induced dysplasia and hepatocellular carcinoma. *Hepatology*, *45*(4), 938-947. https://doi.org/10.1002/hep.21622

Yusenko, M. V., Kuiper, R. P., Boethe, T., Ljungberg, B., van Kessel, A. G., & Kovacs, G. (2009). High-resolution DNA copy number and gene expression analyses distinguish chromophobe renal cell carcinomas and renal oncocytomas. *BMC Cancer*, *9*, 152. https://doi.org/10.1186/1471-2407-9-152
